# Transcriptome dynamics of the microRNA inhibition response

**DOI:** 10.1093/nar/gkv603

**Published:** 2015-06-18

**Authors:** Jiayu Wen, Elenora Leucci, Roberto Vendramin, Sakari Kauppinen, Anders H. Lund, Anders Krogh, Brian J. Parker

**Affiliations:** 1 The Bioinformatics Centre, Department of Biology, University of Copenhagen, Ole Maaloes Vej 5, 2200 Copenhagen N, Denmark; 2 Biotech Research and Innovation Centre (BRIC), University of Copenhagen, Ole Maaloes Vej 5, 2200 Copenhagen N, Denmark; 3 Laboratory for Molecular Cancer Biology, Center for the Biology of Disease, VIB, 3000 Leuven, Belgium; Laboratory for Molecular Cancer Biology, Center of Human Genetics, VIB, 3000 Leuven, Belgium; 4 Department of Haematology, Aalborg University Hospital, A.C. Meyers Vnge 15, 2450 Copenhagen SV, Denmark; 5 Bioinformatics Institute, Agency for Science, Technology and Research (A*STAR), 30 Biopolis street, #07-01, Singapore 138671

## Abstract

We report a high-resolution time series study of transcriptome dynamics following antimiR-mediated inhibition of miR-9 in a Hodgkin lymphoma cell-line—the first such dynamic study of the microRNA inhibition response—revealing both general and specific aspects of the physiological response. We show miR-9 inhibition inducing a multiphasic transcriptome response, with a direct target perturbation before 4 h, earlier than previously reported, amplified by a downstream peak at ∼32 h consistent with an indirect response due to secondary coherent regulation. Predictive modelling indicates a major role for miR-9 in post-transcriptional control of RNA processing and RNA binding protein regulation. Cluster analysis identifies multiple co-regulated gene regulatory modules. Functionally, we observe a shift over time from mRNA processing at early time points to translation at later time points. We validate the key observations with independent time series qPCR and we experimentally validate key predicted miR-9 targets. Methodologically, we developed sensitive functional data analytic predictive methods to analyse the weak response inherent in microRNA inhibition experiments. The methods of this study will be applicable to similar high-resolution time series transcriptome analyses and provides the context for more accurate experimental design and interpretation of future microRNA inhibition studies.

## INTRODUCTION

MicroRNAs (miRNA) are small RNAs providing a post-transcriptional regulatory system which, when base-pairing to a target gene, lead to message degradation and/or translational repression. miRNA transfection and inhibition have been widely used to study miRNA target genes in various systems. However, the majority of these studies were based on one or two time points, e.g. (1,2) and the dynamics of the time series response following miRNA inhibition has not been previously well studied. Here we report a high resolution time series study of transcriptome dynamics following locked nucleic acid (LNA) anti-miR mediated inhibition of miR-9 in a Hodgkin lymphoma (HL) cell-line at 17 time points over 112 h. We investigate both dynamic aspects of the miRNA post-transcriptional response, including downstream interaction over time with transcriptional regulatory systems, as well as changes in function induced by the miR-9 inhibition response. The entire high resolution time-series was repeated in total across four biological replicate samples: we used a microarray dataset to produce a full transcriptomic analysis for the overall exploratory analysis on one sample, with three replicate quantitative polymerase chain reaction (qPCR) time courses to validate the results of specific findings from the exploratory analysis.

In a previous study (3), we applied functional data analytic (FDA) statistical analyses (4) to a miR-124 transfection dataset at a low time resolution (seven time points). We found evidence of a multi-phasic response to miRNA transfection, with direct miRNA targets showing an initial early downregulation of mRNA levels and with apparent additional downregulation responses at ∼32 to 72 h, which we hypothesized were due in part to coherent indirect regulation. A limitation of this study was that the super-physiological levels of transfected miRNA may have not been representative of typical physiological responses over time and transfection raised the possibility of off-target effects. Also, the small number of time samples limited the sensitivity to detect the response.

The current study is the first high resolution time series study following miRNA inhibition. It utilizes a tiny LNA (locked nucleic acid) anti-miR oligonucleotide, complementary to the 5′ end seed region of miR-9 (5), which forms a high affinity duplex with miR-9 within the RISC complex (6), functionally blocking its activity and leading to de-repression of miR-9 target mRNAs (7,8). Thus miRNA inhibition gives insight into a more physiological response pattern of miRNA regulation and biological targets, without off-target effects.

miR-9 is a conserved miRNA with three paralogous genes (*hsa-mir-9-1, hsa-mir-9-2* and *hsa-mir-9-3*), reviewed in (9). Although miR-9 was originally characterized as a brain-expressed miRNA (10), it is expressed in multiple tissues, e.g. it is involved in immune regulation (11–14) and is amongst the 10 most abundant miRNAs in the HL cell line used in this study (15–17). miR-9 was chosen for this experiment as it is involved in key pathways and in several disease processes, and is a well-studied miRNA across multiple tissues with a substantial literature including examples of validated direct targets and regulatory interactions. The miR-9 LNA inhibition system in the cell line used in this study has been previously validated (5).

*Direct targets* represent genes directly bound by miR-9-loaded RISC complex and are here defined by predictions using AGO-CLIP-Seq data; *indirect targets* are defined as genes showing a downstream response to miR-9 inhibition but which are not themselves directly targeted by miR-9. In this study we show that the dynamic response to miRNA inhibition involves an initial direct de-repression response by 4 h, earlier than previously reported (1) and we give evidence of widespread coherent downstream indirect amplification of this initial direct response at ∼32 h. We use the time series data to predict miR-9 direct targets in HL cell lines and analyse co-regulated modules. The predicted miR-9 direct targets involve multiple roles in post-transcriptional regulatory control, including small RNA processing with *DICER, TNRC6* and *AGO3*; message splicing with *SRSF1*; message degradation with *XRN1*/*XRN2*; P-body message storage with *LSM14A*; and a co-regulatory role by targeting of several RNA binding proteins, including *QKI, PUM2* and *RANBP2*.

## MATERIALS AND METHODS

### Cell culture, transfection and miR-9 inhibition, and RNA extraction

The HL cell line L428, was grown under standard conditions in 5% CO_2_ at 37 °C in RPMI 1640-glutamax (Gibco, Invitrogen) supplemented with 10% FBS (Hyclone, Thermo Scientific). The L428 were transfected via unassisted uptake (18) by adding LNA-antimiR-9 or LNA scramble control oligonucleotides directly to the culture medium at a final concentration of 10 μM. Total RNA was obtained by TRIZOL (Invitrogen) extraction. For miR-9 inhibition the following LNA oligonucleotides were used: 5’- AACCAAAG -3’ to target miR-9 (hsa-miR-9-5p miRBase id, matching miR-9 seed base positions 2–9) and 5’- TCATACTA -3’ as a negative control (see (5)). The LNA were checked before the experiment by luciferase with the perfect match reporter. The efficiency and specificity of the miR-9 inhibition was previously verified using the same LNA sequence, cell line and method ((19) Supplementary Figure 1 A): a sample was co-transfected with a (firefly) luciferase reporter containing 2X perfect match miR-9 binding sites and the normalized luciferase activity measured relative to a renilla luciferase control. Comparing the normalized luciferase activity of the LNA sequence against the scramble control sequence showed a relative luciferase activity of ∼5.8. The oligonucleotides were retained in the medium throughout the full time course of the experiment—overall levels would be expected to decrease across the experiment due to uptake by cells and dilution by cell growth. To minimize potential confounding of the dynamic response by synchronized cell-cycle effects, we ensured that the cells were not synchronized by cell cycle during the experiment: the cell line was subcultured 24 h before the LNA inhibition at the initiation of the experiment to ensure that the RNA sampling was during the culture exponential growth phase, where the cells are desynchronized. The HL cell line used here has a doubling time of 35 h, which gives an upper bound on cell cycle generation time.

**Figure 1.**
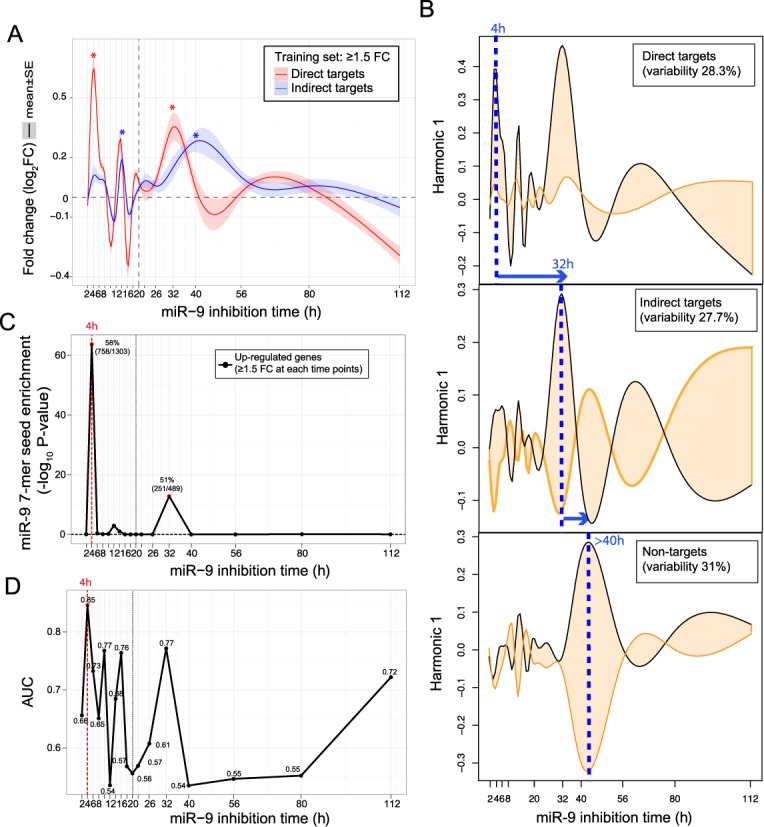
Time-series curves of gene expression fold change (FC) following miR-9 inhibition show a multiphasic response, with initial miR-9-specific direct target response occurring by 4 h. (**A**) Red curve shows the FC response (mean ± SE) for a set of genes computationally enriched for direct miRNA target genes (see ‘Materials and Methods’ section for set definitions). This shows an initial direct inhibition response by 4 h and downstream responses. By contrast, the blue curve shows FC response (mean ± SE) for a set of of genes enriched for indirect miR-9 target genes and does not show a substantial 4 h response. Asterisk denotes highly significantly (*P* < 1E-3) above background variance at other time points. Bands show SEM at each time point. (**B**) Functional PCA of computationally defined sets, from top to bottom: miR-9 direct target set, miR-9 indirect target set, non-target set (see ‘Materials and Methods’ section for set definitions). Shown are the first functional PCA harmonic which is the major variance component. Blue dashed line shows position of first major variance peak. Orange and black lines demarcate the variation limits of the mean when this variance component is added. The three sets show distinct dynamics with the major variance components commencing at progressively later time points for direct, indirect and non-targets, respectively: direct targets show a large asymmetric variance at early time points of 4 h and later at 32 h; indirect targets show predominantly symmetric variance at 32 h and non-targets show a major variance downstream after 40 h. (**C**) Significance of enrichment of miR-9 seeds relative to background, calculated separately for each time point, in genes showing ≥1.5 FC response. There are two statistically significant peaks, with a highly significant enrichment at 4 h, and a smaller, but still highly significant, peak at 32 h. This supports the hypothesis of an initial direct miRNA response at 4 h and a 32 h coherent secondary response. (**D**) Predictive performance of gene expression FC in discriminating miR-9 direct and indirect target gene sets, estimated separately for each time point. The highest predictive performance, measured by AUC of genes ranked by FC at each time point, is at 4 h. Good predictive performance is also seen downstream at 32 h.

*Experimental design:* the full time course used for the microarray transcriptome-wide experiment was: 2, 4, 6, 8, 10, 12, 14, 16, 18, 20, 22, 26, 32, 40, 56, 80, 112 h post-miR-9 inhibition (0 h = LNA added). Each LNA-antimiR treated cell-line sample was matched with an LNA scramble control at each time point. Microarrays were Affymetrix HuGene-1_0-st-v1. This overall time schedule was divided into two intervals using independent cell cultures: one covering early time points (2–20 h) and another covering later time points (22–112 h).

Raw data were submitted to GEO with ID: GSE52710.

### Normalization

All arrays passed standard quality checks. The data were RMA normalized (20) across all arrays simultaneously and log_2_ fold change (FC) against scramble LNA control was computed. As RMA normalization assumes approximately equal distributions across samples and can lead to residual non-linear intensity-dependent biases when this does not hold, especially when there is unequal distribution of up and downregulated genes (21,22), we performed an additional post-normalization step to further reduce inter-array technical differences: a loess fit to the mean curve of the M–A plot of a large set of human house-keeping genes (23) was centred by a non-linear correction of FC (M) depending on mean gene expression (A). Low-expressed genes where the loess fit was imprecise, defined as S.E. <0.02, were not adjusted. This post-normalization step was necessary, as demonstrated by Figure S13 showing a systematic bias at 4 h corrected by the post-normalization. Figure S14 shows MA plots at all time points.

### Definition of model training sets

An initial training set of computationally predicted direct miR-9 targets was defined by the set of predictions with false discovery rate (FDR) <0.2 from the ‘Antar’ PAR-clip-based miRNA target predictive model of (24) (with PhastCons conservation score of the seed ≥0.9). An initial non-target set was defined by the set of genes with no miR-9 seed match (7 or 8-mer) in UTR or CDS and also not predicted as miR-9 targets by the ‘Antar’ models of (24) (either the PAR-CLIP or transfection based models) or TargetScanS or PITA predictors. Importantly, the ‘Antar’ miRNA target prediction model that was used to define the initial sets was trained using AGO PAR-clip binding-sites and not trained using expression data, so avoiding possible selection biases confounding the later analyses of differences in expression curve shape between putative targets and non-target gene sets.

Non-specific filtering requiring a FC (absolute FC of ≥1.5) in the up or down direction at any time point was used to define the subset of genes that responded with either direct or indirect expression changes following miR-9 inhibition in HL cells. A final training set of putative *direct targets* for the HL cell line was defined by applying this non-specific filtering to the set of predicted direct miR-9 targets as defined above, giving a training set size of *N* = 161. A corresponding putative *indirect target* set (i.e. genes showing some downstream response to miR-9 inhibition, but not themselves directly targeted by miR-9) was defined by applying the same non-specific filtering to the non-target set above, and then subsampling to match the size of the direct training set. A putative *non-target* set representing genes showing no substantial direct or indirect response was defined by genes that did not pass the non-specific filter above (i.e. not showing an absolute FC of ≥1.5 in the up or down direction at any time point) and that were not predicted miR-9 targets. It was then subsampled to be the same size as the direct target set.

### Functional data analysis

After normalization, the time series FC samples were converted to continuous functions by spline-fitting: we used a set of 19 B-spline basis functions of order 4 (for cubic smoothing splines). Knots were located at the data points; additional regularized smoothing, using a second derivative roughness penalty (*λ* = 0.05) was applied (4).

The data were then subjected to an unsupervised, exploratory data analysis using functional principal component analysis (PCA) (3–4,25) and a graph-based functional clustering method (26). Functional PCA decomposes the overall complex variance of each time course into separate, simpler, ‘eigenfunction’ components ranked by fraction of variance explained. The functional PCA sets were defined as above (except using a non-specific filtering FC threshold of ≥1.2 to ensure adequate set sizes). For the functional clustering and functional predictive models, the individual curves were additionally scaled to have the same root mean square and then the functional inner product performed between the first derivative of the FC curves. This method was designed to focus the analysis purely on curve shape, to avoid confounding of the analysis by overall per-gene differences in expression and to avoid biasing the analysis toward high differential expression genes only.

For a supervised predictive analysis, the computationally defined direct and indirect miR-9 target sets as defined above were used as training sets (except using a non-specific filtering absolute FC threshold of ≥1.2 to ensure adequate training set sizes). Functional non-parametric predictive models were based on a k-nearest neighbour classifier using the functional inner product of the first derivative curves. Separate models were built for different phases of the response: early models trained on 2–10 and 2–20 h and a late model trained on 22–112 h, as well as a model trained on all time points. The predictive performance of the trained models was tested by 10-fold cross-validation (CV) with area under the ROC curve (AUC) as performance measurement (AUC is a measure of predictive performance, ranging from 1.0 to 0.5 for perfect to random prediction, respectively). Predicted miR-9 targets of this model were defined as those with a high posterior probability (≥0.75) and predicted miR-9 non-targets defined as those with low posterior probability (≤0.25). Predicted miR-9 targets were then ranked by FC at 4h. FDR estimates for the predictive models were estimated by a shuffling procedure: the fraction of false positives was estimated by applying the same procedure to null sets defined by random sampling of genes using exactly the same non-specific filtering cutoffs and parameters as for the real model.

To estimate the specificity of the miR-9 model, it was compared against 2–10 h early models similarly trained using pooled ‘Antar’ miRNA target prediction sets for all non-miR-9 miRNAs with prediction set size ≥150. Members of these non-miR-9 training sets showing miR-9 or miR-9* 7-mer seed matches in UTR or CDS were excluded, to minimize confounding by the effects of genes co-targeted by miR-9. The final pooled non-miR-9 training sets were randomly subsampled to match the size of the miR-9 training sets and the predictive performance (AUC) of the models compared.

### Enrichment analyses

Gene set enrichment analyses were performed using R/Bioconductor (27). Gene sets were defined using the following resources: miRNA seed and TF motif target genes sets from MSigDB v 3.0 (28); RNA-binding protein (RBP) target gene sets from (29) (see supplementary Table S2 for details); TF gene sets from (30); RNA binding protein gene sets from doRiNA (31); immune gene sets from http://www.immport.org; ARE set from ARED (32). A set of highly significantly enriched TF motifs and miR seeds was defined as those that showed significant GSEA (*P* < 1E-4) at any time point.

To analyse downstream targets of TFs, two subsets of TFs were formed: those that are predicted targets of miR-9 using the FDA predictive model; and those that are predicted non-targets of miR-9. Corresponding TF target gene sets were defined for these two TF subsets, based on MSigDB v 3.0 (28), with target gene sets combined for all TFs within each subset and TF target genes intersecting both subsets excluded.

### Experimental validation

To validate the reproducibility of the expression response across independent biological replicates, time series qPCR of selected predicted miR-9 direct target genes and control non-target genes was performed across three independent biological replicate cell cultures following LNA-antimiR-9 inhibition using the identical protocol to the microarray experiment, with identical sequences for the LNA-antimiR and scramble controls. Life Technologies Fast Sybr Master Mix and qPCR kit were used for qPCR; and high-capacity cDNA Reverse Transcription Kit for RT, according to manufacturer's instructions; 1 μg RNA was used for the reverse transcriptions, with RNA pooled from the biological replicates. The geometric mean of three housekeeping genes—*GAPDH, ACTB* and *YWHAQ*—selected from amongst the genes showing lowest FC variance across all microarray time points and with no 7-mer miR-9 seeds in UTR or CDS, were used in the denominator of the ΔΔCt normalization (33), with no correction for primer efficiency differences. The time schedule for the qPCR was 1, 2, 3, 4, 5, 6, 8, 10, 12, 14, 16, 18, 20, 22, 26, 32, 40 h, with a higher time resolution at the early time points than the microarray experiment (3 and 8 h were subsequently excluded based on quality filtering). All qPCRs were also repeated in three technical replicates. Statistical significance of the 2–4 h peak FC in comparison with the background level of gene activity across time was computed by one sample *t*-test of mean FC from 2 to 4 h against mean FC of time points from 5 to 40 h.

To validate the predicted miR-9 targets, an affinity purification (‘pull-out’) approach was used: low levels (2 nM) of a biotin-tagged and thiouridine-labelled miR-9 were transfected into the L428 HL cells, then *in* *vivo* cross-linked, then miR-9 with its target messages was purified using streptavidin beads. qPCR (*n* = 3) was used to measure the enrichment of the target transcripts in biotin-tagged and thiouridine-labelled miR-9 compared to non-thiouridine-labelled controls, as in (5).

## RESULTS AND DISCUSSION

The transcriptome-wide expression of antimiR-9 treated HL samples was measured from 2 to 112 h post-inhibition. Each LNA-antimiR treated cell-line sample was matched with an LNA scramble control. The FC of the antimiR-9 samples relative to the corresponding scramble LNA controls was computed for all annotated genes at each time point and used in the following analyses. The sampling schedule spacing was increasing with time, 2, 4, 6, 8, 10, 12, 14, 16, 18, 20, 22, 26, 32, 40, 56, 80 and 112 h, giving higher time resolution at early time points and coarser resolution sampling at later time points to capture critical early events (34). A transcriptome-wide analysis of this time series dataset, Figure 1, revealed the following general features of the inhibition response of miR-9.

### Multiphasic time-course response is indicative of a multi-layered regulatory process

We first used a non-tissue-specific computational miRNA target predictor, ‘Antar’ (24), based on seed and flanking sequence features, but not expression, with rigorous cutoffs to define a small (*N* = 161) but high confidence training set of predicted miR-9 direct targets. This training set of putative direct targets was used both for an initial exploratory data analysis to investigate curve shape differences between direct and indirect (causatively downstream) targets, and, later, to train a predictive model specific to the HL cell line used, based on the time-series expression data (see ‘Materials and Methods’ section for formal set definitions).

As previously reported, experimental miRNA perturbations typically lead to only moderate, e.g. ≈1.5, FC in expression (1–2,6,8,35). Moreover, miRNA inhibition typically leads to much smaller FC than miRNA over-expression, e.g. three times lower FC (log_2_) reported for let-7b (1), and our miR-9 inhibition data were consistent with this. We required a minimum absolute FC threshold of 1.5 at any time point to define a gene as showing some response to the miRNA perturbation in the following exploratory analyses.

Figure 1 A shows the mean response curve over (predicted) miR-9 direct targets compared with (predicted) indirect targets. The mean miR-9 direct target curve showed two major peaks: an initial substantial upward peak at ∼4 h (FC = 1.58) and a subsequent upward peak at ∼32 h (FC = 1.28). These peaks were significantly above the background variation across other time points (4 h: *P* = 8E-9, 32 h: *P* = 9E-4; *t*-test). There were also smaller intermediate perturbations at ∼14–16 h. By contrast, for predicted non-miR-9 targets, the mean indirect target curve showed no substantial 4 h peak (FC = 1.08, *P* = 0.35) compared with direct targets and a delayed downstream peak at 32–40 h (*P* = 7E-8). We hypothesize that this multiphasic response is due to an initial direct miR-9 response at ≤4 h, leading to the upward de-repression response expected of a miRNA inhibition, followed by later, causally downstream, indirect responses.

This miR-9 inhibition multi-phasic response over time is broadly similar to the miR-124 transfection response reported in (3), indicative of a complex multi-stage regulatory cascade (Figure S1). However, a major difference in this study is that the response to antimiR-9 inhibition appears as sharply delineated peaks, compared with the more diffuse response of miR-124 transfection. We conjecture that the super-physiological concentrations of miR-124 transfection lead to sustained induced degradation, whereas the competitive inhibition by antimiR-9 in this study perturbs the physiological levels of miRNA, in which case cellular homeostatic mechanisms likely act to return to pre-existing levels more quickly after the initial perturbation, causing a sharp initial impulse response.

To sensitively reveal overall differences in gene responses, we used functional PCA (3) to give a high level decomposition into variance components of the dynamic responses of the direct, indirect and non-target sets of genes (Figure 1 B). These three sets showed strikingly different dynamics progressively: direct targets showed a large asymmetric variance at 4 and 32 h, indirect targets showed a delayed major symmetric variance at 32 h and later, and non-targets showed a major variance component much later, after 40 h. These progressively delayed variance components support that the 4 h response represents a direct miRNA effect and the 32 h response represents a downstream secondary response and beyond 40 h represents further delayed downstream perturbations.

### Direct target de-repression response occurs by 4 h

We further analysed the initial major peak occurring at 4 h after miR-9 inhibition, earlier than previously reported. Several lines of evidence support that this peak represents a direct miRNA post-transcriptional response. First, miR-9 7-mer seeds are significantly enriched at two time points: 4 and 32 h, with the most significant enrichment at 4 h, compared with background (58 versus 35%; *P*-value = 1.8E-64, binomial test), and with a lesser but still significant enrichment at 32 h (51%; *P*-value = 1.5E-13) (Figure 1 C). Second, we measured how well gene expression FC can distinguish miR-9 direct and indirect target sets at each time point, measured by AUC (area under the ROC curve). We found that FC can distinguish miR-9 direct and indirect target sets maximally at 4 h, with an AUC of 0.82 compared to <0.74 at other time points (Figure 1 D), demonstrating that the initial and most predictive miR-9 inhibition response occurs within the first 4 h.

### 32 h peak represents secondary transcriptional coherent responses

Several lines of evidence support that the later 32 h peak represents an indirect transcriptional response secondary to the initial miRNA perturbation.

**Figure 2.**
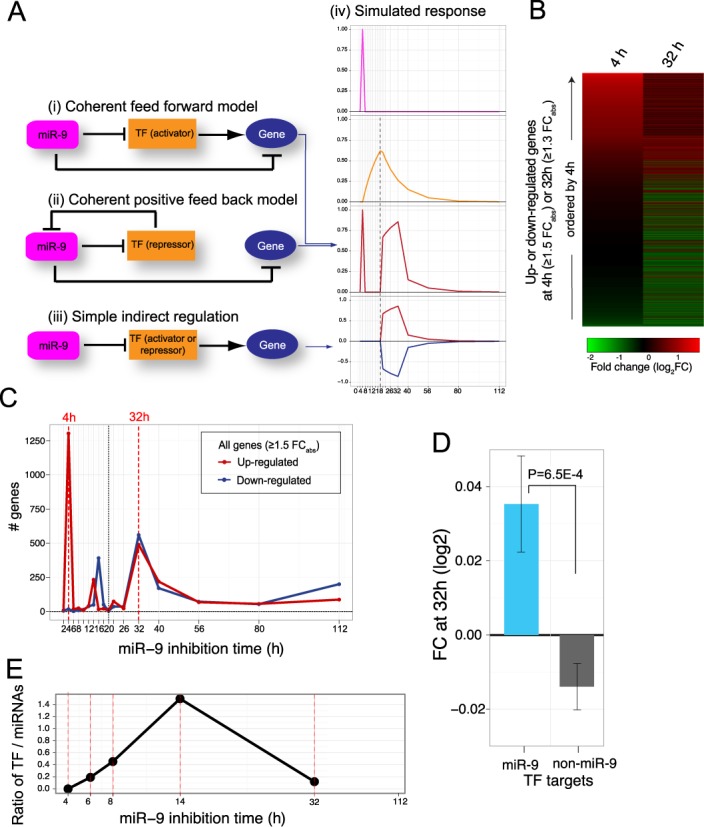
Evidence for downstream 32 h peak representing a coherent secondary response. (**A**) Coherent feed-forward and feed-back models. In the feed-forward model, (i), miR-9 directly targets a gene and also indirectly represses the same gene by targeting an (activator) transcription factor (TF) controlling that gene. In the positive feed-back model, (ii), miR-9 directly targets a gene and also indirectly represses the same gene by targeting a repressor TF that targets the miR-9 genes themselves. Such regulatory mechanisms can potentially amplify and sustain the miR-9 direct response. By contrast, (iii) shows simple downstream indirect regulation of a gene without feed–back or feed-forward regulation. (iv) shows simulated time series response of the various models. Top panel shows initial expression impulse response at 4 h due to LNA de-repression of miR-9; second panel from top shows increased TF (protein) levels due to miR-9 perturbation; third panel shows expression level of the target gene in coherent feed–forward or feed–back regulation, with a 4 h peak due to the direct miR-9 de-repression and a secondary peak commencing once the TF (gold) reaches an activation threshold (dotted line—0.6 in this case); the bottom panel shows the response for the simple indirect regulation of a non-miR-9 target downstream gene (red = activator TF, blue = repressor TF). x-axis = time in hours; y-axis = normalized protein or RNA levels. (**B**) Heatmap showing the distribution of genes with substantial FC response at 4 or at 32 h (absolute FC ≥ 1.5 at 4 h or ≥ 1.3 at 32 h), highlighting that substantial 4 h responses tend to be followed by upward responses at 32 h (red = induced; green = repressed; rows sorted by 4 h response). (**C**) Number of up and downregulated genes with absolute FC ≥1.5 in antimiR-9 treated cells at each time point. The peak at 4 h is asymmetric, with predominantly upregulated genes; the peak at 32 h is symmetric, with a mix of up and downregulated genes, consistent with a TF-driven secondary response at this time. (**D**) Comparison of downstream FC response at 32 h of TF target genes regulated by TFs predicted to be directly targeted by miR-9 (blue) compared with FC response of targets of TFs predicted not to be miR-9 direct targets (grey), The large positive response for TFs targeted by miR-9 is consistent with coherent feed–forward regulation (analysis based on genes showing substantial response, ≥1.5 absolute FC, at any time point; error bars show SE). (**E**) The ratio of number of highly significantly enriched (*P* < 1E-4; GSEA) transcription factor motifs to miRNA seeds across time. Four hours shows significantly enriched miRNA seed motifs but no significant TF motifs; 14 h shows substantial enrichment of TF motifs relative to miRNA seeds; 32 h shows substantial enrichment of both (time points showing no significant miRNA or TF motif enrichment not shown).

Regulatory control systems can be analysed in terms of their simplest fundamental regulatory components and include feed-forward and feed-back mechanisms (36–39). *Coherent feed-forward* loops (FFL) occur when a regulator, e.g. miRNA, changes the expression of a target through a direct effect as well as through an indirect path via another regulator in the same direction (Figure 2 A(i)). Coherent FFL can allow a small initial control signal, such as a miRNA direct response, to be converted into a larger longer-lasting response (e.g. a transcriptional response) (40–42). In particular, a gene regulated by both a fast post-transcriptional mechanism as well as a slower translational mechanism can provide an overall faster and more precise response than a simple single target mechanism. This fits with the concept of miRNAs as a fine-tuning mechanism of gene expression, with overall levels being sustained by larger downstream transcriptional responses and with the direct initial response used for fast fine-tuning of expression levels (2,35,43). In previous general computational studies of regulatory systems, it has been noted that FFL are more common than expected by chance (44,45) and there are published examples of such coherent feed-forward regulation whereby an initial miRNA effect is enhanced by a later transcriptional response in the same direction (40,45–47). *Feedback* regulation (Figure 2 A(ii)) occurs when a target transcription factor affects the transcription of the regulator itself. Positive feedback is amplifying (40), whereas negative feedback is typically stabilizing.

In the regulatory model we hypothesize, miR-9 targets a range of genes directly at 4 h and for these genes the initial post-transcriptional impulse response is amplified and sustained downstream, at 32 h, by a coherent feed-forward or feed-back transcriptional mechanism to give a sustained biological effect, see Figure 2 A(i) and (ii). Additionally, by 32 h additional genes will be secondarily regulated by simple indirect regulation by miR-9 targetted TFs initiated at 4 h, see Figure 2 A (iii), and these indirect responses will be a mix of up and downregulation depending on whether activator or repressor TFs were involved.

Figure 2 A(iv) shows the results of simulating the feed-forward and feed-back models. The TF dynamic responses were modeled by the basic dynamic equation of (41,48), $\frac{dY}{dt}=\beta -\alpha \cdot Y$, with production rate α and degradation rate β for TF *Y* set to approximately match the experimental results, with decay half life defined to be 10 h. The top panel shows the initial expression impulse response at 4 h due to LNA de-repression of miR-9. The second panel shows transiently increased TF (protein) levels due to this miR-9 perturbation. The third panel shows the expression level of the target gene, consisting of a 4 h peak due to the direct miR-9 de-repression and a secondary peak commencing once the TF accumulates to reach an activation threshold for the target gene promoter (defined as 0.6 in this simulation), sufficient to regulate the target gene. This causes a delay (dotted line): for a TF, an upper bound on this delay is typically on the order of one cell cycle generation time (40). By contrast, the bottom panel shows the response for a simple indirect regulation of a non-miR-9 target downstream gene by activator or repressor TFs: as it is not a miR-9 target it does not show the 4 h de-repression peak but does show the delayed downstream response.

For the experimental data, the heatmap (Figure 2 B and Figure S5) shows genes having substantial up (red) or down (green) response at either 4 h (≥1.5 absolute FC) or at 32 h (≥1.3 absolute FC) (*N* = 5278), demonstrating that 4 h responses do indeed tend to be upregulated and to be followed by upregulation responses at 32 h. To quantify this, of the 2196 upregulated genes at 32 h, highly significantly more genes were overlapped by preceding upregulation at 4 h than expected by chance (28% overlap, *P* = 9E-8, Fisher test). This demonstrates that the 32 h upward peak is predominantly a downstream indirect response, as opposed to an alternative hypothesis in which it represented a direct miRNA response of a subset of genes for which the de-repression was somehow delayed until 32 h, in which case such delayed genes would not be predicted to show a preceding 4 h response. Conversely, when a 32 h response does follow a 4 h direct miRNA response, it is overwhelmingly coherent i.e. in the same upward direction: among the 1042 genes showing both upregulation at 4 h (≥1.5 FC) and up or downregulation at 32 h (≥1.3 absolute FC), almost all genes (98%) were upregulated at 32 h. These results are consistent with the response curves, Figure 2 A(i) and (ii), of the hypothesized model.

When comparing the number of genes showing an absolute FC ≥1.5 up or downregulation at each time point (Figure 2 C): the 4 h response shows a marked asymmetry with predominantly upregulated genes (1303 up, 17 down; *P* = 2E-16, binomial test of proportion difference > 10%), characteristic of a direct post-transcriptional miRNA response, whereas the 32 h time point shows a symmetric mix of up and downregulation (489 up, 560 down; *P* = 0.9, binomial test of proportion difference > 10%), more consistent with causally downstream indirect transcriptional activator/repressor regulation i.e. due to a mix of Figure 2 A(i), (ii) and (iii) type responses. A similar result is seen in Figure S3 which is based on all genes showing a substantial response ≥1.5 up- or down-FC at any time point.

Both coherent feed-forward, Figure 2 A(i), and coherent feed-back, A(ii), are plausible mechanisms contributing to the observed 32 h peak. According to the coherent feed forward model, TF genes that are part of such FFL will be transcriptional activators and will be miR-9 targets, as will the targets of their TF product. To investigate this further, we divided transcription factor target genes into two sets: those for which their corresponding TF gene is predicted to be directly targeted by miR-9, compared with those for which their corresponding TF gene is predicted not to be directly targeted by miR-9 (see ‘Materials and Methods’ section). If coherent FFLs are a prominent mechanism then by the FFL model we would expect the target gene sets of TFs that are themselves targeted by miR-9 to have an enrichment for miR-9 direct targets. By contrast, TF genes that are not miR-9 targets cannot be part of such a coherent FFLs, and so the target gene sets of these would not be expected to be enriched for miR-9 direct targets. Indeed, we observed weakly significant enrichment of predicted miR-9 direct targets amongst the TF targets of miR-9 controlled compared with non-miR-9 controlled TFs (22.1 versus 18.0%; *P* = 0.03). We then compared downstream FC responses of the targets of these two sets of transcription factors. The largest FC difference was observed at 32 h with a significant upregulation (activator) response for genes targeted by miR-9 regulated TFs, compared to a smaller downregulation for genes controlled by non-miR-9 regulated TFs (Figure 2 D). These results are consistent with coherent feed-forward regulation being a contributing regulatory mechanism of miR-9 in the HL cell line. A candidate TF potentially involved in coherent feed-back mechanisms involving miR-9 is the repressive transcription factor REST (aka NRSF) (Figure S4), which is involved in validated examples of positive feed-back involving miR-9 in other tissues. Notably, it is a predicted miR-9 direct target and is known to regulate miR-9 promoters to cause transcriptional gene silencing by histone acetylation; miR-9-1 and miR-9-2 promoters have been reported as REST targets in human and all three paralogues in mouse (49–51).

As noted above, transcription factor and miRNA interaction plays a major role in downstream coherent responses, so we sought to further analyse gene co-expression changes over time in TF and miRNA targets following miR-9 inhibition. We performed motif gene set enrichment analyses (GSEA) based on ranking by FC at each time point and found many significantly enriched known TF motifs and miRNA seed motifs. To estimate the degree of significant miRNA target enrichment compared with transcription factor target enrichment, we calculated the ratio of the number of highly significant (*P* < 1E-4) TF motifs and miRNA seeds from the GSEA at each time point (Figure 2 E and Figure S11). At 4 h we observed a significant enrichment for only miRNA seeds and no enriched TF motifs, as expected for perturbation of direct miRNA targets at this time point. At 14 h we saw a substantial enrichment of TF motifs relative to miRNA seeds, suggesting a first level of downstream secondary responses of TF targets of miR-9 direct targets at this time. By 32 h enrichment of both TF motifs and miRNA seeds was observed, consistent with a later downstream coherent indirect response.

Taken together, the miR-9 inhibition results in this experimental system indicate an early, ≤4 h, direct miRNA effect followed by secondary indirect perturbations, with predominantly coherent transcriptional regulation of the same direct target genes later by ∼32 h, likely involving both feed-forward and feed-back mechanisms.

### miR-9 target prediction model trained on transcriptome dynamics

We next built a predictive model utilizing the high-resolution time series data to identify miR-9 targets which are functional in the HL cell-line. The high time-resolution of this study allowed for the full utilization of FDA techniques which can analyse the curve shape, and in particular changes in curve derivatives over time (3), as features to increase performance and robustness of the predictive model. Such power is required to distinguish the smaller perturbations induced by miRNA inhibition.

This required an initial training set for which we used the ‘Antar’ direct and indirect sets defined above. Note that the ‘Antar’ CLIP-seq-based predictive model of (24) is a general non-tissue-specific predictor, which was trained using miRNA target and contextual sequence features from AGO CLIP-seq target sites and so did not use expression data for training. By contrast, the FDA model incorporates as a feature the additional information provided by the time series expression data, allowing for the training of a more sensitive prediction model specific for the HL cell-line. This provides a more complete survey of miR-9 targets in HL than is possible with a non-tissue specific genomic sequence-based miRNA target predictor.

**Figure 3.**
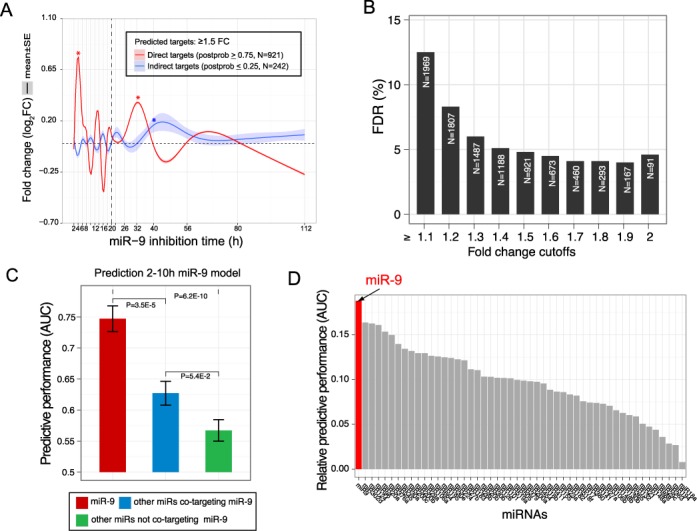
miR-9 predictive model trained on time series responses. (**A**) FC curves (mean ± SE) of high confidence predicted direct targets (≥0.75 posterior probability) with FC ≥1.5-fold versus predicted indirect targets (≤0.25 posterior probability, black). Predicted direct miR-9 targets show an early 4 h peak and a 32 h upregulatory coherent response, compared with no substantial response for predicted indirect miR-9 targets (grey). Asterisk denotes peaks highly significantly, *P* < 1E-3, above background variance across all other time points. Bands show SEM at each time point. (**B**) Number of predicted miR-9 direct targets with estimated false discovery rate at various FC thresholds at 4 h for the miR-9 prediction model. (**C**) Specificity of miR-9 predictive model. Performance of the miR-9 FDA model (red) was compared to two models trained on other miRNAs: miRNAs sharing substantial co-targets with miR-9 (Antar predicted target overlap >15%, blue) and miRNAs without substantial co-targets with miR-9 (Antar predicted target overlap <8%, green). The miR-9 model shows a much better performance compared with the non-miR-9 models, giving evidence that the response is miR-9 specific. The increased AUC of the non-miR-9 miRNAs showing computational evidence of miR-9 co-targeting (blue) to those without (green) is consistent with the AUC increase of the blue set being due to computationally undetected miR-9 targets. Error bars show SE over 10 CV iterations. *P*-values calculated by Hanley–McNeil estimation. (**D**) A model trained for miR-9 performs better than equivalent models trained for other miRs. Prediction performance of models trained identically for all miRNAs with >150 predicted targets. The y-axis estimates the model specificity by the increase in predictive performance (AUC) for the miRNA for which the predictive model was built, relative to the mean AUC for other miRNAs pooled. The predictive model for miR-9 is the most specific, demonstrating that the direct target response seen at 4 h is miR-9 specific. Other highly ranked miRNAs suggest co-targetting with miR-9.

Although genes showing a larger FC are likely of biologically importance, lower FC targets can also have a biologically substantial effect, for example by acting mainly by blocking translation (1) or by simultaneously targeting several members of a key pathway (36). We aimed to build a predictive model able to classify miR-9 target genes even with low overall FC. This is particularly important for miRNA perturbation studies which typically have moderate FC. To do so we trained the model by extracting curve shape only and normalizing away overall amplitude of response. This ensured maximal sensitivity of the model to overall response curve shape differences in the FDA analysis, which avoided biasing prediction towards only target genes with high FC responses. We defined high-confidence predicted direct targets of the FDA predictive model as those with a prediction posterior probability of ≥0.75 and defined high-confidence predicted indirect targets by a posterior probability of ≤ 0.25. We then ranked these predicted targets by the FC at 4 h and thresholded at a defined lower bound on FC to form the final prediction set (Figure 3 B).

To focus on the initial direct target de-repression response at ∼4 h, we trained the FDA predictive model using only early time points from 2 to 20 h. The overall cross-validated AUC for this early FDA predictive model was 0.76 (S.E. 0.02), while a model built using all time points gave a similar AUC of 0.77 (S.E. 0.02), demonstrating that the majority of the signal to distinguish direct and indirect miR-9 targets is within the first 10 h. Further, the good predictive performance of the FDA model validates that the initial training sets were of high quality.

Consistent with the mean FC curves based on the training sets in Figure 1 A, the mean FC response curve over the predicted high confidence miR-9 direct targets from this FDA model (posterior probability ≥ 0.75) showed peaks at 4 and 32 h significantly above background levels, in contrast to the high confidence indirect targets (posterior probability ≤ 0.25) which did not show a significant peak at 4 h (Figure 3 A). This result was robust to varying the FC threshold from 1.5- to 2-fold, which did not change the overall target response curve shape, see Supplementary Figure S2B.

Comparing the predicted targets from an early model trained on 2–20 h with a late model trained on 22–112 h, we observed a substantial 49% overlap, again supporting that the majority of miR-9 direct targets are followed by a coherent downstream response.

We further wanted to test the specificity of the FDA predictive model for miR-9. Figure 3 C shows that the predictive performance of the trained miR-9 FDA model was substantially higher for predicting miR-9 targets compared to a model predicting the targets of all other (non-miR-9) miRNAs pooled, indicating a miR-9 specific model. Figure 3 D shows this increase in AUC for the miRNA used to train the model, relative to a background AUC over all other miRNAs, for predictive models trained independently for each miRNA showing a substantial number (*N* ≥ 150) of predicted Antar targets. Notably, the specificity of the miR-9 FDA predictive model was the highest amongst all miRNA FDA models. Note that the training and testing pipeline was repeated identically for each of these non-miR-9 miRNAs, demonstrating in an unbiased manner that the time series responses seen in the data are indeed a specific consequence of the miR-9 inhibition and not due, for example, to a non-specific effect on miRNA response. Notably, several non-miR-9 miRNAs showed a predictive performance above random levels which we hypothesize is a result of co-targeting of genes by both miR-9 and multiple other miRNAs.

### Predicted miR-9 targets in HL cell line indicate roles in RNA processing and RBP regulation

To generate a complete list of predicted functional miR-9 targets specific for the HL cell line, we applied the trained time-series FDA predictive model above to all genes. In total, the early 2–20 h FDA model predicted 921 miR-9 targets with posterior probability >0.75 and FC ≥1.5 at 4 h.

We estimated the FDR for the direct target predictions using a shuffling approach, comparing with random background within this sample (see Methods). Figure 3 B shows the FDR and number of predicted targets for various FC cutoffs. An FC cutoff of 1.5 gave an FDR of <5%. Figure S6 shows the miR-9 seed enrichment of the predicted miR-9 direct target sets at 4 h across varying FC cutoffs: all cutoffs show highly significant miR-9 seed enrichment.

Of 33 published miR-9 targets validated across multiple cell lines from several previous studies (Table S1), 13 were predicted as functional targets in this HL cell line. These included e.g. *REST* which has been shown to be involved in a negative feedback loop involving REST silencing complex in brain (50); and *ALCAM* which is regulated by miR-9 in a negative auto-regulatory loop in hepatoma, possibly affecting cell migration (52).

Several predicted targets are involved in post-transcriptional regulation at multiple stages of RNA processing including: *DICER1* (5), *AGO3 (EIF2C3)* and *TNRC6B* which are involved in miRNA function; *SRSF1, PRPF4B* and *NSRP1* which are involved in alternative splicing; *XPO4* which is involved in the nuclear export of miRNA precursors; *XRN1/2* which are involved in RNA degradation; *LSM14A* which is involved in message storage in P-bodies; and *EIF5* which is involved in translation. Seventy-eight predicted targets were RBP genes, including *QKI* which is involved in RNA processing including splicing; *RANBP2* which is involved in nuclear transport; and *STAU1* which is involved in STAU1-mediated mRNA decay. A total of 148 predicted targets were transcription factor (TF) genes, including *STAT4, ONECUT2, ELK4* and *POU2F1 (aka OCT2)*, shown to be involved in lymphocyte biology (53). Other notable targets include those involved in cell cycle regulation, such as *CENPF* and *CEP152*; DNA repair e.g. *BRCA1*; and the mTOR pathway e.g. *RICTOR, FBXO9*. See Supplementary Table S1 for a full list of predicted targets.

### Replicated time series qPCR confirms early miR-9 direct response

**Figure 4.**
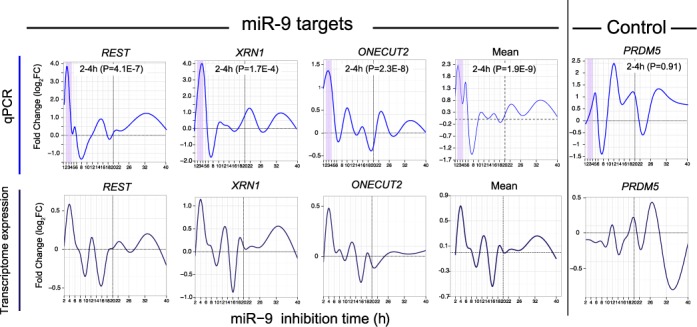
Time series qPCR of selected positive and negative example genes. The time schedule for the qPCR was from 1 to 40 h with a higher hourly time resolution at the early time points. qPCR over three pooled biological replicate samples was performed using the identical miR-9 inhibition protocol to the transcriptome–wide study. The response of the three predicted miR-9 direct target genes *REST, XRN1* and *ONECUT2* and the mean of these (top panel) qualitatively matched the major features of the response seen in the transcriptome–wide analysis (bottom panel), with an initial large peak at 2–4 h and a mean upward peak at approximately 32 h. The mean FC from 2 to 4 h (purple highlight) of *REST, XRN1* and *ONECUT2* was significantly higher than the background mean FC variance over the rest of the time course from 5 to 40 h (*t*-test). The qPCR analysis reveals that the early direct response peak may initiate as early as 2–4 h. The negative control gene *PRDM5* by contrast did not show a dominant peak at 2–4 h and the mean FC over 2–4 h was not significantly higher than over 5–40 h.

To maximize the sensitivity of the FDA exploratory data analysis for the fixed number of microarrays available, the transcriptome-wide experimental design used above was optimized to maximize time resolution against number of biological replicates, see (34). To validate the reproducibility of the measured curve response across independent biological replicates, time series qPCR of selected positive and negative example genes over three pooled biological replicate samples was performed using the identical miR-9 inhibition protocol (Figure 4). The time sample schedule for the qPCR was 1, 2, 3, 4, 5, 6, 8, 10, 12, 14, 16, 18, 20, 22, 26, 32, 40 h which gave finer hourly time resolution at early time-points. The response curves of the three predicted miR-9 direct target genes *REST, XRN1* and *ONECUT2*, and the mean over these, qualitatively matched the major features of the response detected in the transcriptome analysis, with an initial large impulse response peak at ≤4 h significantly above background variation across other time points (mean curve *P* = 2E-9; *t*-test) and an upward peak at ∼32 h significantly above background (mean curve *P* = 5E-5; *t*-test), particularly pronounced for *REST*. The negative control gene *PRDM5*, by contrast, did not show a substantial peak at ≤4 (*P* = 0.9) or 32 h (*P* = 0.2). This time-series qPCR analysis on selected target genes indicates that the initial miRNA response may occur as early as 2–4 h.

### Experimental validation of selected predicted miR-9 targets

**Figure 5.**
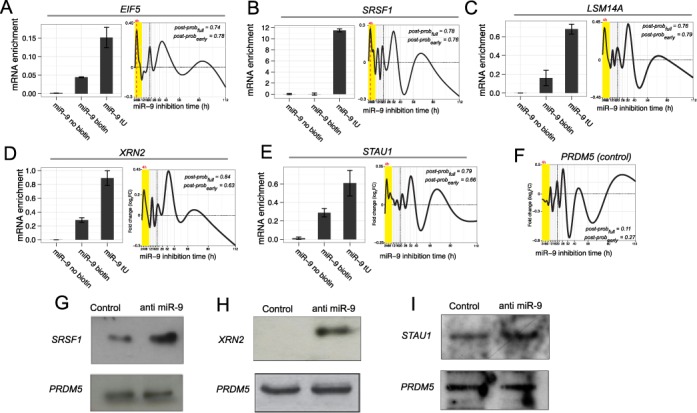
Experimental validation of selected predicted miR-9 targets: *EIF5, SRSF1, LSM14A, XRN2* and *STAU1. PRDM5* is a predicted non-miR-9 target as a control. (**A**–**E**) shows miR-9 pull-out validation. Biotin-tagged and thiouridine-labelled miR-9 was transfected into the L428 HL cells and qPCR used to measure their ability to pull-down the corresponding messages, qPCR was normalized to input (see ‘Materials and Methods’ section for details). ‘miR-9 tU’: miR-9 labelled with biotin and containing 4-thiouridine, leading to pull-down; ‘miR-9 biotin’: miR-9 labelled with biotin only, as control; ‘miR-9 no biotin’: miR-9 only, as control. Error bars show SEM (*n* = 3). The corresponding mean dynamic response curves are shown (yellow highlights the 2–10 h early time points). *EIF5, SRSF1, LSM14A, XRN2* and *STAU1* show significant miR-9 tU pull-down evidence of miR-9 targeting compared to controls with biotin only or without biotin and the time series curves show the expected peak at 4 h. By contrast, the negative control *PRDM5* time series curve (**F**) does not show a characteristic peak at 4 h. (**G**–**I**) shows western blots for *SRSF1, XRN2* and *STAU1* validating translational induction compared with the scramble control; the non-target *PRDM5* by contrast shows no substantial change relative to control.

Several computationally predicted targets involving key RNA processing steps were experimentally validated: *SRSF1, LSM14A* and *EIF5, XRN2, STAU1*. We used an affinity purification approach, pull-out validation, in which biotinylated and thiouridine-labelled miR-9 was transfected into the HL cells and qPCR used to measure their ability to pull-down the corresponding messages (see ‘Materials and Methods’ section). This method has been used previously to validate targets of several miRNAs (5). We showed that biotin-tagged and thiouridine-labelled miR-9 can efficiently pull-out *XRN2, SRSF1, STAU1, LSM14A* and *EIF5* compared to non-thiouridine-labelled controls, validating them as direct miR-9 targets (Figure 5 A–E). Mean dynamic response curves of these target genes showed the expected peak at 4 h. Using western blots, we further confirmed that miR-9 efficiently regulates *XRN2, SRSF1* and *STAU1* at a translational level while non-target *PRDM5* did not show substantial change relative to control (Figure 5 G–I). *PRDM5* is a predicted non-target and did not show a FC peak at 4 h as expected (Figure 5 F).

### Functional cluster analysis detects miR-9 regulatory modules

We next aimed to identify gene modules co-regulated by miR-9 with similar dynamic responses. We used a functional cluster analysis approach utilizing the full time series curves, with the first derivative as a feature vector and we identified 135 functional clusters with ≥10 genes (15 large clusters with ≥ 50 genes).

**Figure 6.**
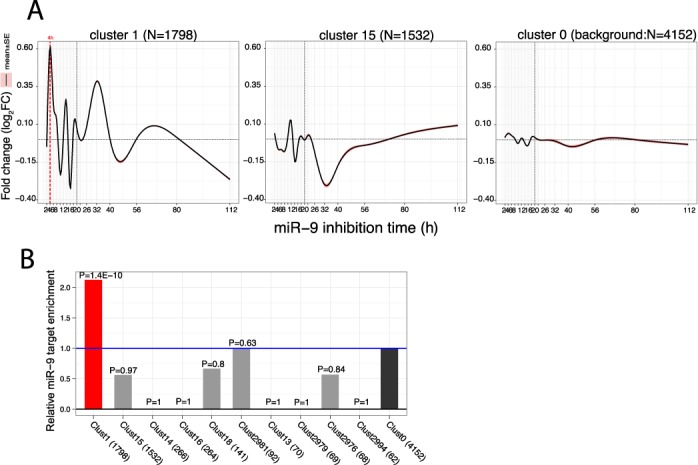
Functional clustering reveals distinct regulatory modules with varying dynamic response. (**A**) Mean FC curves for the two largest functional clusters. Cluster 1 has a similar response to that of direct miR-9 targets and is highly enriched for miR-9 seeds. Cluster 15 is significantly enriched for targets of the repressive TF REST/NRSF, showing a repressive response at 32 h. The background cluster shows the mean curve of genes not otherwise classified into regulatory modules and shows no substantive response, demonstrating no substantial normalization artifacts between microarrays. Bands show standard error at each curve position. (**B**) miR-9 target enrichment in each cluster. To avoid possible confounding by transcript length differences, we compared miR-9 3’UTR target enrichment of each cluster against a subsample of the background cluster of the same size, with sampled genes matched to have the same 3’UTR length distribution (mean of of 10 repetitions was reported). Cluster 1 is highly enriched for miR-9 targets, whereas the other clusters show equality or depletion relative to background (blue line denotes background enrichment of 1.0).

The largest cluster (cluster 1; *N* = 1798) consisted of primarily miR-9 direct target genes. Genes in this cluster showed substantial upregulation at 4 h and at 32 h (Figure 6 A), with a similar response to miR-9 direct targets and showed a highly significant miR-9 seed enrichment in both 3′UTR (2.1X; *P* = 1.4E-10) and CDS (1.5X; *P* = 2E-9) compared to the background (Figure 6 B). Functionally, the highest gene ontology (GO) or KEGG pathway enrichment was for cell cycle (mitotic) functions (*P* = 3.3E-11); ubiquitin-mediated proteolysis (*P* = 5E-6); and DNA repair (*P* = 2E-16). An enrichment analysis for targets of RBPs using CLIP-seq datasets (see Materials and Methods’ section) showed that this cluster is strikingly enriched for targets of multiple RBPs predicted as miR-9 direct targets including SRSF1 (*P* = 8E-105); AGO2-3 (*P* = 2E-66); TIAL1 (*P* = 8E-48); QKI (*P* = 4E-40); PUM2 (*P* = 2E-32); TIA1 (*P* = 6E-13). This suggests that RBPs may play a co-regulatory role in the miR-9 regulatory mechanism. This cluster is also highly enriched for AU rich elements (ARE) (*P* = 7E-5) which are often found in 3′UTRs of cytokines and other immune early response genes.

The second largest cluster (cluster 15; *N* = 1532) is, functionally, enriched for proteinaceous extracellular matrix (*P* = 3E-9); cell adhesion (*P* = 8E-5); NCAM1 cell adhesion mediated interactions (*P* = 9E-5); and cell junctions (*P* = 4E-5). Notably, Hodgkin lymphoma is associated with sclerosis and fibroblast infiltration and miR-9 has previously been reported to be involved in focal adhesion pathways in collagen (54). This cluster showed a substantial negative FC at 32 h (Figure 6 A), which we hypothesize is a downstream simple transcriptional repressive response. This cluster was the only cluster showing significant enrichment for targets of transcriptional repressor REST (*P* = 2E-4).

The background ‘cluster’ (cluster 0; *N* = 4152), defined as otherwise unclustered genes, had a miR-9 seed proportion at background levels (38%) and showed a flat mean approximately centred at 0 as expected, supporting that there were no substantial per-chip normalization artifacts confounding the analyses above. Additional time series mean FC curves for example clusters are shown in Figure S7. A full table of clusters listing gene members is given in Supplementary Table S3; a table of cluster gene set enrichments is given in Supplementary Table S4; additional cluster descriptions are given in supplementary results.

### Dynamic functional genomics of miR-9 inhibition in HL cell line

#### miR-9 seeds in coding regions lead to substantial message degradation response

**Figure 7.**
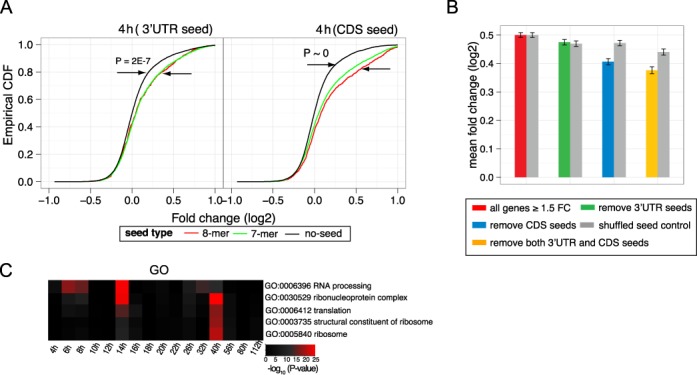
(**A** and **B**) CDS miR-9 targets show a substantial degradation response. (A) Cumulative distribution, at 4 h, of FC for genes with one or more 3′UTR or CDS miR-9 seeds, compared with genes with no miR-9 seeds, demonstrating an enrichment of miR-9 seeds in both CDS and 3′UTR. (B) Decrease in FC on removing genes from an initial set of all genes showing ≥1.5 FC at 4 h (red). The genes excluded were those containing miR-9 7-mer seeds in: 3′UTR (green), CDS (blue) and whole gene body (yellow), respectively. These were compared with controls (grey) which used a shuffled miR-9 seed sequence. Notably, removing CDS seed sites showed a substantial decrease in FC relative to randomized controls, while removal of 3′UTR seeds was not greater than random control. Note that the randomized control seed sets also show a small systematic decrease in FC which is likely due to systematic dependencies between FC and UTR/CDS length. (**C**) Heatmap of gene ontology (GO) functional enrichment based on expression FC across time. Red = statistical significance. There is a functional shift from mRNA processing at early time points to translational terms at later time points. GO terms were included where highly significant enrichment with *P*-value < 1E-7 occurred at any time point (see Figure S12 for full heatmap).

miRNA targets in coding regions (CDS) have been shown to be extensive in AGO CLIP–seq studies (55) and in some cases to have substantial effect on expression level (56). Several recent studies have suggested that CDS sites have a less substantial effect on message degradation compared with UTR sites (57). To quantify the impact of CDS miR-9 target sites on our analysis, we analysed the effect of CDS and UTR miR-9 target sites for the size of the degradative response. Figure 7 A shows the FC cumulative distribution, at 4 h, for genes with one or more miR-9 seeds in 3′UTR or CDS, compared with genes with no miR-9 seeds. Both 3′UTR and CDS seeds show a marked enrichment in up-regulated genes. Notably, CDS miR-9 seeds are more highly enriched than 3′UTR seeds (3′UTR: 8-mer versus no seed: KS statistic = 0.12 *P* = 2E-7; CDS: 8-mer versus no seed: KS statistic = 0.19; *P* ≈ 0)(see Supplementary Figures S8 and S9 for similar plots at all time points).

As 4 h demonstrates the major direct target response, we further compared the FC at 4 h following sequential removal of targets with 7-mer miR-9 seeds in 3′UTR, CDS and both (Figure 7 B). We noted that FC is weakly positively correlated with 3′UTR length (Spearman correlation coefficient = 0.27; *P* < 2E-16). As a longer UTR is more likely to match a given seed sequence by chance, to avoid possible confounding with sequence length FC differences were compared with control analyses which used randomly shuffled miR-9 miRNA sequence. Surprisingly, the expression decrease on removing genes with CDS seeds was substantially larger than random control, while removing genes with 3′UTR seeds was not greater than random control, demonstrating that inhibition of miR-9 CDS seeds in HL cell-line has a large effect on message degradation. Removing genes with both 3′UTR and CDS seeds showed the largest message degradation. Consequently, we have incorporated both CDS and 3′UTR seeds in relevant analyses above.

#### GO enrichments show a progression from RNA processing to translational terms

To investigate gene functional changes across time following the miR-9 perturbation, we further performed GO analysis independently at each time point based on FC. This revealed two highly enriched terms: RNA processing at early time points, with peak at 14 h, and translation and ribosomal-related terms at later time points, at ∼40 h (Figure 7 C and Supplementary Figure S12). This gene function shift from RNA processing at early time points to protein synthesis terms at later time points is consistent with a regulatory cascade showing indirect downstream protein synthesis responses secondary to upstream RNA processing perturbations. Significant changes in alternative splicing patterns of genes involving the mTOR pathway were also evident downstream, see Supplementary Figure S10.

## CONCLUSIONS

We performed a high-resolution time series study of miR-9 inhibition in an HL cell-line which revealed physiological aspects of the complex miRNA regulatory response over time.

Dynamically, it revealed a multi-phasic response showing a very early direct response within the first 4 h, enriched for miR-9 seeds, followed by substantial downstream indirect responses at ∼32 h. This was further confirmed by time series qPCR. These responses are consistent with coherent feed-forward or feed-back regulation being a typical mechanism of miRNA regulation, amplifying the initial perturbation into larger sustained downstream responses, involving transcriptional, and likely RBP-mediated post-transcriptional, regulation. As evidence of this early direct response, a predictive model trained on early data points ≤10 h could clearly distinguish a miR-9-specific signal. Both this study and our earlier miR-124 study (3) have provided evidence for a downstream coherent response, suggesting that such downstream amplification of an initial miRNA perturbation may be a common feature of miRNA transcriptome responses.

Functionally, this study revealed a changing physiological response across time in response to miR-9 inhibition, with the overall functional profile shifting from transcriptional to translational processing. Target prediction revealed that miR-9 is involved in regulating multiple stages of the RNA life-cycle and post-transcriptional regulatory mechanisms such as RBPs. Several key targets were validated by biotin-tagged thiouridine-labelled miR-9 pull-out and western blots. Functional clustering revealed large miR-9-controlled regulatory modules involved in cell adhesion and other functions.

Methodologically, we developed sensitive FDA predictive methods designed to analyse the intrinsically weak signal of miRNA inhibition studies. Using a high-resolution time series design allowed for more sensitive and specific predictive models to be built than possible with single time-point studies, with FDR estimates demonstrating a strong signal above noise background. Functional cluster analysis revealed a new catalogue of miR-9 driven regulatory modules in the HL cell-line showing specific functions and functional predictive models provided a set of miR-9 targets in the studied HL cell line. The methods of this study will be applicable to high-resolution time series expression analyses of other miRNAs and the increased understanding of the dynamics following miRNA inhibition revealed by this study provides the context for more accurate experimental design and interpretation of future miRNA inhibition studies.

## SUPPLEMENTARY DATA

Supplementary Data are available at NAR Online.

SUPPLEMENTARY DATA

## References

[1] Selbach M., Schwanhäusser B., Thierfelder N., Fang Z., Khanin R., Rajewsky N. (2008). Widespread changes in protein synthesis induced by microRNAs. Nature.

[2] Baek D., Villén J., Shin C., Camargo F. D., Gygi S. P., Bartel D.P. (2008). The impact of microRNAs on protein output. Nature.

[3] Parker B.J., Wen J. (2009). Predicting microRNA targets in time-series microarray experiments via functional data analysis. BMC Bioinformatics.

[4] Ramsay J.O., Wickham H., Graves S., Hooker G. FDA: Functional Data Analysis (2011) R package version 2.2.7. http://www.cran.r-project.org/package=fda.

[5] Leucci E., Zriwil A., Gregersen L.H., Jensen K.T., Obad S., Bellan C., Leoncini L., Kauppinen S., Lund A.H. (2012). Inhibition of miR-9 de-represses HuR and DICER1 and impairs Hodgkin lymphoma tumour outgrowth in vivo. Oncogene.

[6] Elmén J., Lindow M., Silahtaroglu A., Bak M., Christensen M., Lind-Thomsen A., Hedtjärn M., Hansen J.B., Hansen H.F., Straarup E.M. (2008). Antagonism of microRNA-122 in mice by systemically administered LNA-antimiR leads to up-regulation of a large set of predicted target mRNAs in the liver. Nucleic Acids Res..

[7] Stenvang J., Petri A., Lindow M., Obad S., Kauppinen S. (2012). Inhibition of microRNA function by antimiR oligonucleotides. Silence.

[8] Obad S., dos Santos C.O., Petri A., Heidenblad M., Broom O., Ruse C., Fu C., Lindow M., Stenvang J., Straarup E.M. (2011). Silencing of microRNA families by seed-targeting tiny LNAs. Nat. Genet..

[9] Yuva-Aydemir Y., Simkin A., Gascon E., Gao F.-B. (2011). MicroRNA-9: functional evolution of a conserved small regulatory RNA. RNA Biol..

[10] Farh K. K.-H., Grimson A., Jan C., Lewis B.P., Johnston W.K., Lim L.P., Burge C.B., Bartel D.P. (2005). The widespread impact of mammalian microRNAs on mRNA repression and evolution. Science.

[11] Anderson P. (2008). Post-transcriptional control of cytokine production. Nat. Immunol..

[12] Taganov K.D., Boldin M.P., Baltimore D. (2007). MicroRNAs and immunity: tiny players in a big field. Immunity.

[13] Tsitsiou E., Lindsay M.A. (2009). MicroRNAs and the immune response. Curr. Opin. Pharmacol..

[14] Katsanou V., Papadaki O., Milatos S., Blackshear P.J., Anderson P., Kollias G., Kontoyiannis D.L. (2005). HuR as a negative posttranscriptional modulator in inflammation. Mol. Cell.

[15] Nie K., Gomez M., Landgraf P., Garcia J.-F., Liu Y., Tan L. H.C., Chadburn A., Tuschl T., Knowles D.M., Tam W. (2008). MicroRNA-mediated down-regulation of PRDM1/Blimp-1 in Hodgkin/Reed-Sternberg cells: a potential pathogenetic lesion in Hodgkin lymphomas. Am. J. Pathol..

[16] Gibcus J.H., Tan L.P., Harms G., Schakel R.N., de Jong D., Blokzijl T., Möller P., Poppema S., Kroesen B.-J., van den Berg A. (2009). Hodgkin lymphoma cell lines are characterized by a specific miRNA expression profile. Neoplasia.

[17] Van Vlierberghe P., De Weer A., Mestdagh P., Feys T., De Preter K., De Paepe P., Lambein K., Vandesompele J., Van Roy N., Verhasselt B. (2009). Comparison of miRNA profiles of microdissected Hodgkin/Reed-Sternberg cells and Hodgkin cell lines versus CD77+ B-cells reveals a distinct subset of differentially expressed miRNAs. Br. J. Haematol..

[18] Stein C.A., Bo Hansen J., Lai J., Wu S. (2010). Efficient gene silencing by delivery of locked nucleic acid antisense oligonucleotides, unassisted by transfection reagents. Nucleic Acids Res.

[19] Leucci E., Patella F., Waage J., Holmstrøm K., Lindow M., Porse B., Kauppinen S., Lund A.H. (2013). microRNA-9 targets the long non-coding RNA MALAT1 for degradation in the nucleus. Sci. Rep..

[20] Irizarry R.A., Hobbs B., Collin F., Beazer-Barclay Y.D., Antonellis K.J., Scherf U., Speed T.P. (2003). Exploration, normalization, and summaries of high density oligonucleotide array probe level data. Biostatistics.

[21] Freudenberg J., Boriss H., Hasenclever D. (2004). Comparison of preprocessing procedures for oligo-nucleotide micro-arrays by parametric bootstrap simulation of spike-in experiments. Methods Inf. Med..

[22] Pelz C.R., Kulesz-Martin M., Bagby G., Sears R.C. (2008). Global rank-invariant set normalization (GRSN) to reduce systematic distortions in microarray data. BMC Bioinformatics.

[23] Eisenberg E., Levanon E.Y. (2003). Human housekeeping genes are compact. Trends Genet..

[24] Wen J., Parker B.J., Jacobsen A., Krogh A. (2011). MicroRNA transfection and AGO-bound CLIP-seq data sets reveal distinct determinants of miRNA action. RNA.

[25] Coffey N., Hinde J. (2011). Analyzing time-course microarray data using Functional Data Analysis—a review. Stat. Appl. Genet. Mol. Biol..

[26] Hartuv E., Shamir R. (2000). A clustering algorithm based on graph connectivity. Information Processing Letters.

[27] Luo W., Friedman M.S., Shedden K., Hankenson K.D., Woolf P.J. (2009). GAGE: generally applicable gene set enrichment for pathway analysis. BMC Bioinformatics.

[28] Liberzon A., Subramanian A., Pinchback R., Thorvaldsdóttir H., Tamayo P., Mesirov J.P. (2011). Molecular signatures database (MSigDB) 3.0. Bioinformatics.

[29] Anders G., Mackowiak S.D., Jens M., Maaskola J., Kuntzagk A., Rajewsky N., Landthaler M., Dieterich C. (2012). doRiNA: a database of RNA interactions in post-transcriptional regulation. Nucleic Acids Res..

[30] Fulton D.L., Sundararajan S., Badis G., Hughes T.R., Wasserman W.W., Roach J.C., Sladek R. (2009). TFCat: the curated catalog of mouse and human transcription factors. Genome Biol..

[31] Cook K.B., Kazan H., Zuberi K., Morris Q., Hughes T.R. (2011). RBPDB: a database of RNA-binding specificities. Nucleic Acids Res..

[32] Bakheet T., Williams B.R.G., Khabar K.S.A. (2006). ARED 3.0: the large and diverse AU-rich transcriptome. Nucleic Acids Res..

[33] Hellemans J., Mortier G., De Paepe A., Speleman F., Vandesompele J. (2007). qBase relative quantification framework and software for management and automated analysis of real-time quantitative PCR data. Genome Biol..

[34] Bar-Joseph Z., Gitter A., Simon I. (2012). Studying and modelling dynamic biological processes using time-series gene expression data. Nat. Rev. Genet..

[35] Krützfeldt J., Rajewsky N., Braich R., Rajeev K.G., Tuschl T., Manoharan M., Stoffel M. (2005). Silencing of microRNAs in vivo with ‘antagomirs’. Nature.

[36] Ebert M.S., Sharp P.A. (2012). Roles for microRNAs in conferring robustness to biological processes. Cell.

[37] Brandman O., Meyer T. (2008). Feedback loops shape cellular signals in space and time. Science.

[38] Osella M., Bosia C., Corá D., Caselle M. (2011). The role of incoherent microRNA-mediated feedforward loops in noise buffering. PLoS Comput. Biol..

[39] Cohen S.M., Brennecke J., Stark A. (2006). Denoising feedback loops by thresholding–a new role for microRNAs. Genes Dev..

[40] Alon U. (2007). Network motifs: theory and experimental approaches. Nat. Rev. Genet..

[41] Mangan S., Alon U. (2003). Structure and function of the feed-forward loop network motif. Proc. Natl. Acad. Sci. U.S.A..

[42] Nazarov P.V., Reinsbach S.E., Muller A., Nicot N., Philippidou D., Vallar L., Kreis S. (2013). Interplay of microRNAs, transcription factors and target genes: linking dynamic expression changes to function. Nucleic Acids Res..

[43] Bartel D.P., Chen C.-Z. (2004). Micromanagers of gene expression: the potentially widespread influence of metazoan microRNAs. Nat. Rev. Genet..

[44] Tsang J., Zhu J., van Oudenaarden A. (2007). MicroRNA-mediated feedback and feedforward loops are recurrent network motifs in mammals. Mol. Cell.

[45] Zhou Y., Ferguson J., Chang J.T., Kluger Y. (2007). Inter- and intra-combinatorial regulation by transcription factors and microRNAs. BMC Genomics.

[46] Litvak V., Ramsey S.A., Rust A.G., Zak D.E., Kennedy K.A., Lampano A.E., Nykter M., Shmulevich I., Aderem A. (2009). Function of C/EBPdelta in a regulatory circuit that discriminates between transient and persistent TLR4-induced signals. Nat. Immunol..

[47] Breving K., Esquela-Kerscher A. (2010). The complexities of microRNA regulation: mirandering around the rules. Int. J. Biochem. Cell Biol..

[48] Alon U. (2007). An Introduction to Systems Biology: Design Principles of Biological Circuits, Vol.10 of Chapman & Hall/CRC Mathematical and Computational Biology Series.

[49] Conaco C., Otto S., Han J.-J., Mandel G. (2006). Reciprocal actions of REST and a microRNA promote neuronal identity. Proc. Natl. Acad. Sci. U.S.A..

[50] Packer A.N., Xing Y., Harper S.Q., Jones L., Davidson B.L. (2008). The bifunctional microRNA miR-9/miR-9* regulates REST and CoREST and is downregulated in Huntington's disease. J. Neurosci..

[51] Laneve P., Gioia U., Andriotto A., Moretti F., Bozzoni I., Caffarelli E. (2010). A minicircuitry involving REST and CREB controls miR-9-2 expression during human neuronal differentiation. Nucleic Acids Res..

[52] Wang J., Gu Z., Ni P., Qiao Y., Chen C., Liu X., Lin J., Chen N., Fan Q. (2011). NF-kappaB P50/P65 hetero-dimer mediates differential regulation of CD166/ALCAM expression via interaction with microRNA-9 after serum deprivation, providing evidence for a novel negative auto-regulatory loop. Nucleic Acids Res..

[53] Corcoran L., Emslie D., Kratina T., Shi W., Hirsch S., Taubenheim N., Chevrier S. (2014). Oct2 and Obf1 as facilitators of B:T cell collaboration during a humoral immune response. Front. Immunol..

[54] Gaidatzis D., van Nimwegen E., Hausser J., Zavolan M. (2007). Inference of miRNA targets using evolutionary conservation and pathway analysis. BMC Bioinformatics.

[55] Hafner M., Landthaler M., Burger L., Khorshid M., Hausser J., Berninger P., Rothballer A., Ascano M., Jungkamp A.-C., Munschauer M. (2010). Transcriptome-wide identification of RNA-binding protein and microRNA target sites by PAR-CLIP. Cell.

[56] Schnall-Levin M., Rissland O.S., Johnston W.K., Perrimon N., Bartel D.P., Berger B. (2011). Unusually effective microRNA targeting within repeat-rich coding regions of mammalian mRNAs. Genome Res..

[57] Hausser J., Syed A.P., Bilen B., Zavolan M. (2013). Analysis of CDS-located miRNA target sites suggests that they can effectively inhibit translation. Genome Res..

