# Supplementary material for: Transcriptome dynamics of the microRNA inhibition response
Source: Nucleic Acids Res. 2015 Jun 18;43(13):6207–21. doi: 10.1093/nar/gkv603 (PMC4513874; doi:10.1093/nar/gkv603)
Supplement: SUPPLEMENTARY DATA [file supp_43_13_6207__index.html]

Transcriptome dynamics of the microRNA inhibition response — SUPPLEMENTARY DATA 

# Transcriptome dynamics of the microRNA inhibition response

## SUPPLEMENTARY DATA

**Files in this Data Supplement:**

- SUPPLEMENTARY DATA
- SUPPLEMENTARY DATA
- SUPPLEMENTARY DATA
- SUPPLEMENTARY DATA
- SUPPLEMENTARY DATA
