# Supplementary material for: Transcriptome dynamics of the microRNA inhibition response
Source: Nucleic Acids Res. 2015 Jun 18;43(13):6207–21. doi: 10.1093/nar/gkv603 (PMC4513874; doi:10.1093/nar/gkv603)
Supplement: SUPPLEMENTARY DATA [file supp_gkv603_nar-00720-y-2015-File007.pdf]

Supplementary for Transcriptome dynamics of the microRNA inhibition response

Jiayu Wen [1,2,6], Elenora Leucci [2,4,6], Roberto Vendramin [4], Sakari Kauppinen [3], Anders H. Lund [2], Anders Krogh [1,2], Brian J. Parker [1,5,7 *]

[1]The Bioinformatics Centre, Department of Biology and [2] the Biotech Research and Innovation Centre (BRIC), University of Copenhagen, Ole Maaloes Vej 5, 2200 Copenhagen N, Denmark
[3]Department of Haematology, Aalborg University Hospital, A.C. Meyers Vnge 15, 2450 Copenhagen SV, Denmark
[4]VIB Laboratory for Molecular Cancer Biology, University of Leuven, Belgium
[5]Bioinformatics Institute, A*Star, 30 Biopolis St, Singapore

*[6]Contributed equally. [7]To whom correspondence should be addressed: bparker@binf.ku.dk; anders.lund@bric.ku.dk; krogh@binf.ku.dk.
© The Author(s)
This is an Open Access article distributed under the terms of the Creative Commons Attribution Non-Commercial License (http://creativecommons.org/licenses/by-nc/2.0/uk/) which permits unrestricted non-commercial use, distribution, and reproduction in any medium, provided the original work is properly cited.

SUPPLEMENTARY TABLES

Table 1. Published miR-9 targets. Including tissue/cell line where it was experimentally validated, assay used for validation, and references to the report where validated. This list excludes targets validated only from high-throughput methods i.e. microarray or CLIP–seq.

gene	cell type	reference	validation
PRDM1	Hodgkin lymphoma	Nie et al. [22]	other
BCL6	immunity	Zhang et al. [39]	other
NFKB1	monocytes	Asirvatham et al. [2]	reporter assay
POU2F2	immunity	Forrest et al. [7]	other
ALCAM	hepatoma	Wang et al. [32]	luciferase assay
NR2E1	brain	Denli et al. [6]	other
REST	brain	Packer et al. [24]	other
BACE1	brain	Hébert et al. [9]	reporter assay
FOXP1	spinal cord	Otaegi et al. [23]	
CAMTA1	glioblastoma stem cells	Schraivogel et al. [29]	
NTRK3	human neuroblastoma cells	Laneve et al. [13]	other
CDH1	human mammary epithelial cells	Ma et al. [19]	reporter assay
CDX2	gastric cancer cells	Rotkrua et al. [28]	
RAB34	human gastric carcinoma	Luo et al. [18]	other
FOXO1	endometrial cancer	Myatt et al. [21]	other
STMN1	human embryonic stem cells	Delaloy et al. [5]	reporter assay
SIRT1	human embryonic stem cell	Delaloy et al. [5]	Western blot
CHMP2B	human embryonic stem cell	Delaloy et al. [5]	Western blot
PDGFRB	cardiomyocytes	Zhang et al. [40]	
MYOCD	myocardium	Wang et al. [34]	
MMP13	human cartilage	Jones et al. [10]	other
ONECUT2	pancreatic beta cells	Plaisance et al. [26]	other
NFAT5		Asirvatham et al. [2]	
ETS1	human monoblast leukemia cells	Forrest et al. [7]	other
AP3B1	human breast cancer cell line	Selcuklu et al. [30]	reporter assay
CCNG1	human breast cancer cell line	Selcuklu et al. [30]	reporter assay
LARP1	human breast cancer cell line	Selcuklu et al. [30]	reporter assay
MTHFD1L	human breast cancer cell line	Selcuklu et al. [30]	reporter assay
MTHFD2	human breast cancer cell line	Selcuklu et al. [30]	reporter assay
SRPK1	human breast cancer cell line	Selcuklu et al. [30]	reporter assay
ELAVL1	HL cells and xenograft	[15]	WB, luciferase, IHC and pull-out assay
DICER1	HL cells and xenograft	[15]	WB, luciferase, IHC and pull-out assay
MALAT1	HL cells	[16]	WB, luciferase, IHC and pull-out assay

Table 2. CLIP-seq datasets from [1] used to define RBP gene sets

RBP	reference	cell line	% threshold
QKI	Hafner et al. [8]	HEK	10
PUM2	Hafner et al. [8]	HEK	10
ELAV1/HuR	Mukherjee et al. [20]	HEK	1
AGO1-4	Hafner et al. [8]	HEK	2
AGO2	Kishore et al. [11]	HEK	5
hnRNPC	König et al. [12]	HeLa	5
TIA1	Wang et al. [37]	HeLa	5
TIAL1	Wang et al. [37]	HeLa	2

DESCRIPTION OF SUPPLEMENTARY DATA–FILES

1. Supplementary datafile 1: FDA predictive model posterior probabilities. Tabs give results for early (2–10 hour), late (22–112 hour) and combined (2–112 hour) models. Columns indicate seed matches in CDS and UTR regions. Filename: Supplementary_datafile1.xls

2. Supplementary datafile 2: FDA clustering results. TF and RBP enrichments for each defined cluster ≥ 5 elements. Filename: Supplementary_datafile2.txt

3. Supplementary datafile 3: FDA clustering results. Gene lists for each defined cluster ≥ 5 elements. Filename: Supplementary_datafile3.txt

4. Supplementary datafile 4: FDA clustering results. Gene set enrichments for each defined cluster ≥ 5 elements. Filename: Supplementary_datafile4.txt

SUPPLEMENTARY RESULTS:

Additional functional cluster descriptions:

cluster 16: The fourth largest cluster, (N=264) showed a highly enriched TF target gene set for TFCP2 (p=1E-3). It showed Reactome enrichment for integrin cell surface interactions (p=7E-4) and Kegg enrichment for cell junctions (p=5E-4). It was enriched for cytokine genes (p=6E-3), including interleukin members such as IL-3. It did not show enrichment for particular RBP target genes. It showed a downward peak at 32 hours, suggesting that TFCP2 is acting as a negative regulator for these targets.

cluster 18: The fifth largest cluster (N=141) was highly enriched for the TF ELF28 (p=3E-4). It showed enrichment for antigen processing and presentation immune genes (p=3E-3; Immport set). It was very highly enriched in AGO2* (p=1E-8; PAR-CLIP). KEGG enrichment showed focal adhesion (5E-4) and GO enrichment showed antigen processing and presentation of exogenous peptide antigen (p=4E-4).

cluster 13: Cluster 13 (N=70) has a similar shape to cluster 14, and it is also enriched in splicing-related SFRS1 (aka SF2/ASF) targets (p=4E-4) and to a lesser extent AGO2 (p=0.01). It appears to have somewhat distinct functional enrichments to cluster 13 however with Reactome: golgi associated vesicle biogenesis (p=4E-05) and GO: glycolipid metabolic process (p=6E-05).

cluster 3089: Cluster 3089 (N=37) is enriched in ELAV1 (p=4E-7); IGF2BP (p=1E-5) and QKI* (p=3E-3) RBP targets. The most significant TF target enrichment was for NR3C1* (p=7E-4). It shows GO enrichment for proteolysis (p=3E-4; GO). Apart from cluster 1, cluster 3089 is the only one to show highly significant ARE enrichment (class C3; p=8E-3). Interestingly, the curve shape resembles that of cluster 1, and is enriched above background for miR-9 seeds, although this does not reach statistical significance. This cluster probably represents a smaller cluster of direct targets with an alternative dynamic response. The known TF miR-9 target Onecut-2* noted previously is included in this cluster; it has been shown that in beta-cells miR-9 down-regulates the TF Onecut-2 which, acting as a transcriptional repressor, therefore increasing the expression of Sytl4 (Granuphilin/Slp4) [26] (see next section).

cluster 3611: Cluster 3611 (N=8) similarly has a curve shape resembling cluster 1. It is significantly enriched in miR-9 7-mer seeds (p=0.02), and probably also represents a smaller distinguished cluster of miR-9 direct targets. It is enriched in ELAV1 targets (p=1E-5), and is enriched in tRNA 3’ end processing (p:4E-4) (GO).

cluster 2744: Cluster 2744 (N=18) shows striking enrichment for cytokine genes (p=3E-5); it shows a peak at 12 hours consistent with the enrichment for cytokine genes seen in fig 3 (E), consistent with downstream indirect regulation. It is enriched for transcription factor targets of KLF12* (p=6E-3) and LEF1 (p=7E-3).

cluster 2718: Cluster 2718 (N=34) is enriched in translational functions: Reactome translation (p=5E-5); Kegg ribosome (7E-06); GO translational elongation (3E-05); GO ribosomal subunit (3E-05). Genes known to be coregulated with *TPT1** and *NPM1* are enriched (MSigDB) (*TPT1*; p=6E-06); (*NPM1*; 3E-05). E2F1 transcription factor target motifs are enriched (p=2E-4). It shows a delayed major peak at approximately 40 hours, and so may represent a response to increase translation of downstream regulated genes.

cluster 3118: Cluster 3118 (N=22) has a similar curve response to cluster 18, and is enriched in immunity–related targets with enrichment in natural killer cell cytotoxicity (p=1E-2; Immport) and Kegg: natural killer cell mediated cytotoxicity (p=1E-2).

cluster 3445: Cluster 3445 (N=8) is highly enriched in AGO2 target genes (p=8E-6). It shows functional enrichments in Reactome: RNA polymerase III chain elongation (p=4E-3) and microRNA biogenesis (p=7E-3).

cluster 2967: Cluster 2967 (N=35) is enriched for the VIP/ADCYAP1 pathway (1E-5). *PACAP* is known to be expressed in the lymphoid microenvironment to modulation of innate and adaptive immunity, and inhibits apoptosis of activated T cells, and the VIP/PACAP pathway is known to down–regulate NFκ-beta. Interestingly, *NFKB1* itself is included in this cluster. There is enrichment for the BCR signalling pathway (Biocarta: p=7E-3); additional functional enrichments include: Kegg: apoptosis (p=5E-4); B cell receptor signalling pathway (p=6E-3).

cluster 2981: Cluster 2981 has a similar shape to cluster 2967 and is involved in negative regulation of mast cell apoptosis (GO: p=0.005).

cluster 3185: Cluster 3185 (N=22) is annotated as highly enriched for MTF1 target genes (p=5E-5); the closely related MTF2* is a high confidence miR-9 target.

cluster 3051: Cluster 3051 (N=28) is enriched in cytokine receptors (p=9E-3; immport); functional enrichments include: GO: cytokine binding (1E-5); GO: chromatin (3E-5); Reactome: M G1 transition (9E-05); Reactome: ORC1 removal from chromatin (9E-05).

cluster 2740: Cluster 2740 (N=55) is functionally enriched (Reactome:) for hemostasis (p=1E-4). TF target gene enrichments include: FOXA1 (V$HNF3_Q6) (p=8E-4).

cluster 3054: Cluster 3054 (N=16) peaks at 40 hours. It is enriched in TF motifs for POU2F1* (OCT1) (a known miR-9 target) (p=5E-4), GABPA* (p=7E-3). Top GO terms include: GO:0015785 UDP-galactose transport (0.000804303021162744); GO:0021757 caudate nucleus development (0.0008).

cluster 25: Cluster 25 (N=13) has GABPA* (p=4E-3), GABPB1* (p=5E-3) TF, and PUM2* (p=4E-3) RBP target enrichments. It is enriched for neural development GO terms e.g. rhombomere 4 development (p=1E-3)

cluster 2622: Cluster 2622 (N=8) has GABPA* TF target enrichment (p=8E-4). Top GO term is GO:0008092 cytoskeletal protein binding (0.00050)

cluster 3165: Cluster 3165 (N=6) shows STAT1* TF target (p=1E-3) enrichment.

cluster 3224: Cluster 3224 (N=11) is enriched for numerous miRNA targets (p=3E-4 for miR-488) and also shows enrichment for PUM2* (p=2E-3); ELAVL1 (p=3E-3); and QKI* (p=1E-2) RBP targets. Biocarta: VDR PATHWAY(0.0066).

cluster 3053: Cluster 3053 (N=6) is enriched for GABPB1* TF targets (p=1E-3).

cluster 133: Cluster 10 (N=133) is enriched for TCF12* TF targets (p=2E-3).

cluster 2972: Cluster 2972 (N=30) enriched for ETS1 TF targets (p=2E-3) (a known miR-9 target).

cluster 3007: Cluster 3007 shows an inverted response. It is enriched for TF MTF1 targets (p=2E-4) (it likely represents a transcriptional repression).

cluster 2987: Cluster 2987 (N=21) is enriched for RBP targets of TDP-43 (p=6E-4) and SFR1 4E-3. GO enrichment: spermidine catabolic process (0.00106).

cluster 2763: Cluster 2763 (N=11) is enriched for TF targets: FOXO3* (p=1E-4). Functional enriched for ligand gated channel activity (p=0.0002) and GO: negative regulation of chemokine biosynthetic process (0.00050).

cluster 2976: Cluster 2976 (N=68). Reactome: gene expression (p=3E-3); Enriched for targets of RBP SFRS1* (SF2/ASF) (p=5E-4).

cluster 3203: Cluster 3203 (N=10) is enriched for TF FOXO1* targets (p=4E-3)(a known miR-9 target). FOXO1 is known to be able to act as a transcriptional repressor.

REFERENCES

1. Gerd Anders, Sebastian D Mackowiak, Marvin Jens, Jonas Maaskola, Andreas Kuntzagk, Nikolaus Rajewsky, Markus Landthaler, and Christoph Dieterich. doRiNA: a database of RNA interactions in post-transcriptional regulation. *Nucleic Acids Res*, 40(1):D180–6, Jan 2012. doi: 10.1093/nar/gkr1007.
2. Ananthi J Asirvatham, Christopher J Gregorie, Zihua Hu, William J Magner, and Thomas B Tomasi. MicroRNA targets in immune genes and the Dicer/Argonaute and ARE machinery components. *Mol Immunol*, 45(7):1995–2006, Apr 2008. doi: 10.1016/j.molimm.2007.10.035.
3. J. S. Bonifacino. *Current Protocols in Molecular Biology*, chapter Metabolic labelling with amino acids. John Wiley and Sons, New York, 1998.
4. Cecilia Conaco, Stefanie Otto, Jong-Jin Han, and Gail Mandel. Reciprocal actions of REST and a microRNA promote neuronal identity. *Proc Natl Acad Sci U S A*, 103(7):2422–7, Feb 2006. doi: 10.1073/pnas.0511041103.
5. Celine Delaloy, Lei Liu, Jin-A Lee, Hua Su, Fanxia Shen, Guo-Yuan Yang, William L Young, Kathy N Ivey, and Fen-Biao Gao. MicroRNA-9 coordinates proliferation and migration of human embryonic stem cell-derived neural progenitors. *Cell Stem Cell*, 6(4):323–35, Apr 2010. doi: 10.1016/j.stem.2010.02.015.
6. Ahmet M Denli, Xinwei Cao, and Fred H Gage. miR-9 and TLX: chasing tails in neural stem cells. *Nat Struct Mol Biol*, 16(4):346–7, Apr 2009. doi: 10.1038/nsmb0409-346.
7. A R R Forrest, M Kanamori-Katayama, Y Tomaru, T Lassmann, N Ninomiya, Y Takahashi, M J L de Hoon, A Kubosaki, A Kaiho, M Suzuki, J Yasuda, J Kawai, Y Hayashizaki, D A Hume, and H Suzuki. Induction of microRNAs, mir-155, mir-222, mir-424 and mir-503, promotes monocytic differentiation through combinatorial regulation. *Leukemia*, 24(2):460–6, Feb 2010. doi: 10.1038/leu.2009.246.
8. Markus Hafner, Markus Landthaler, Lukas Burger, Mohsen Khorshid, Jean Hausser, Philipp Berninger, Andrea Rothballer, Manuel Ascano, Jr, Anna-Carina Jungkamp, Mathias Munschauer, Alexander Ulrich, Greg S Wardle, Scott Dewell, Mihaela Zavolan, and Thomas Tuschl. Transcriptome-wide identification of RNA-binding protein and microRNA target sites by PAR-CLIP. *Cell*, 141(1):129–41, Apr 2010. doi: 10.1016/j.cell.2010.03.009.
9. Sébastien S Hébert, Katrien Horré, Laura Nicolaï, Aikaterini S Papadopoulou, Wim Mandemakers, Asli N Silahtaroglu, Sakari Kauppinen, André Delacourte, and Bart De Strooper. Loss of microRNA cluster miR-29a/b-1 in sporadic Alzheimer's disease correlates with increased BACE1/beta-secretase expression. *Proc Natl Acad Sci U S A*, 105(17):6415–20, Apr 2008. doi: 10.1073/pnas.0710263105.
10. S W Jones, G Watkins, N Le Good, S Roberts, C L Murphy, S M V Brockbank, M R C Needham, S J Read, and P Newham. The identification of differentially expressed microRNA in osteoarthritic tissue that modulate the production of TNF-alpha and MMP13. *Osteoarthritis Cartilage*, 17(4):464–72, Apr 2009. doi: 10.1016/j.joca.2008.09.012.
11. Shivendra Kishore, Lukasz Jaskiewicz, Lukas Burger, Jean Hausser, Mohsen Khorshid, and Mihaela Zavolan. A quantitative analysis of CLIP methods for identifying binding sites of RNA-binding proteins. *Nat Methods*, 8(7):559–64, Jul 2011. doi: 10.1038/nmeth.1608.
12. Julian König, Kathi Zarnack, Gregor Rot, Tomaz Curk, Melis Kayikci, Blaz Zupan, Daniel J Turner, Nicholas M Luscombe, and Jernej Ule. iCLIP reveals the function of hnRNP particles in splicing at individual nucleotide resolution. *Nat Struct Mol Biol*, 17(7):909–15, Jul 2010. doi: 10.1038/nsmb.1838.
13. Pietro Laneve, Lucia Di Marcotullio, Ubaldo Gioia, Micol E Fiori, Elisabetta Ferretti, Alberto Gulino, Irene Bozzoni, and Elisa Caffarelli. The interplay between microRNAs and the neurotrophin receptor tropomyosin-related kinase C controls proliferation of human neuroblastoma cells. *Proc Natl Acad Sci U S A*, 104(19):7957–62, May 2007. doi: 10.1073/pnas.0700071104.
14. Pietro Laneve, Ubaldo Gioia, Anastasia Andriotto, Francesca Moretti, Irene Bozzoni, and Elisa Caffarelli. A minicircuitry involving REST and CREB controls miR-9-2 expression during human neuronal differentiation. *Nucleic Acids Res*, 38(20):6895–905, Nov 2010. doi: 10.1093/nar/gkq604.

15. E Leucci, A Zriwil, L H Gregersen, K T Jensen, S Obad, C Bellan, L Leoncini, S Kauppinen, and A H Lund. Inhibition of miR-9 de-represses HuR and DICER1 and impairs Hodgkin lymphoma tumour outgrowth in vivo. *Oncogene*, 31(49):5081–9, Dec 2012. doi: 10.1038/onc.2012.15.
16. Eleonora Leucci, Francesca Patella, Johannes Waage, Kim Holmstrøm, Morten Lindow, Bo Porse, Sakari Kauppinen, and Anders H Lund. microRNA-9 targets the long non-coding RNA MALAT1 for degradation in the nucleus. *Sci Rep*, 3:2535, Aug 2013. doi: 10.1038/srep02535.
17. Arthur Liberzon, Aravind Subramanian, Reid Pinchback, Helga Thorvaldsdóttir, Pablo Tamayo, and Jill P Mesirov. Molecular signatures database (MSigDB) 3.0. *Bioinformatics*, 27(12):1739–40, Jun 2011. doi: 10.1093/bioinformatics/btr260.
18. Hongchun Luo, Hongbin Zhang, Zhenzhen Zhang, Xia Zhang, Bo Ning, Jinjun Guo, Na Nie, Bo Liu, and Xiaoling Wu. Down-regulated miR-9 and miR-433 in human gastric carcinoma. *J Exp Clin Cancer Res*, 28:82, 2009. doi: 10.1186/1756-9966-28-82.
19. Li Ma, Jennifer Young, Harsha Prabhala, Elizabeth Pan, Pieter Mestdagh, Daniel Muth, Julie Teruya-Feldstein, Ferenc Reinhardt, Tamer T Onder, Scott Valastyan, Frank Westermann, Frank Speleman, Jo Vandesompele, and Robert A Weinberg. miR-9, a MYC/MYCN-activated microRNA, regulates E-cadherin and cancer metastasis. *Nat Cell Biol*, 12(3):247–56, Mar 2010. doi: 10.1038/ncb2024.
20. Neelanjan Mukherjee, David L Corcoran, Jeffrey D Nusbaum, David W Reid, Stoyan Georgiev, Markus Hafner, Manuel Ascano, Jr, Thomas Tuschl, Uwe Ohler, and Jack D Keene. Integrative regulatory mapping indicates that the RNA-binding protein HuR couples pre-mRNA processing and mRNA stability. *Mol Cell*, 43(3):327–39, Aug 2011. doi: 10.1016/j.molcel.2011.06.007.
21. Stephen S Myatt, Jun Wang, Lara J Monteiro, Mark Christian, Ka-Kei Ho, Luca Fusi, Roberto E Dina, Jan J Brosens, Sadaf Ghaem-Maghami, and Eric W-F Lam. Definition of microRNAs that repress expression of the tumor suppressor gene FOXO1 in endometrial cancer. *Cancer Res*, 70(1):367–77, Jan 2010. doi: 10.1158/0008-5472.CAN-09-1891.
22. Kui Nie, Mario Gomez, Pablo Landgraf, Jose-Francisco Garcia, Yifang Liu, Leonard H C Tan, Amy Chadburn, Thomas Tuschl, Daniel M Knowles, and Wayne Tam. MicroRNA-mediated down-regulation of PRDM1/Blimp-1 in Hodgkin/Reed-Sternberg cells: a potential pathogenetic lesion in Hodgkin lymphomas. *Am J Pathol*, 173(1):242–52, Jul 2008. doi: 10.2353/ajpath.2008.080009.
23. Gaizka Otaegi, Andrew Pollock, Janet Hong, and Tao Sun. MicroRNA miR-9 modifies motor neuron columns by a tuning regulation of FoxP1 levels in developing spinal cords. *J Neurosci*, 31(3):809–18, Jan 2011. doi: 10.1523/JNEUROSCI.4330-10.2011.
24. Amy N Packer, Yi Xing, Scott Q Harper, Lesley Jones, and Beverly L Davidson. The bifunctional microRNA miR-9/miR-9* regulates REST and CoREST and is downregulated in Huntington's disease. *J Neurosci*, 28(53):14341–6, Dec 2008. doi: 10.1523/JNEUROSCI.2390-08.2008.
25. Brian J Parker and Jiayu Wen. Predicting microRNA targets in time-series microarray experiments via functional data analysis. *BMC Bioinformatics*, 10: S32, 2009. doi: 10.1186/1471-2105-10-S1-S32.
26. Valérie Plaisance, Amar Abderrahmani, Véronique Perret-Menoud, Patrick Jacquemin, Frédéric Lemaigre, and Romano Regazzi. MicroRNA-9 controls the expression of Granuphilin/Slp4 and the secretory response of insulin-producing cells. *J Biol Chem*, 281(37):26932–42, Sep 2006. doi: 10.1074/jbc. M601225200.
27. Mark D Robinson and Terence P Speed. Differential splicing using whole-transcript microarrays. *BMC Bioinformatics*, 10:156, 2009. doi: 10.1186/ 1471-2105-10-156.
28. Pichayanoot Rotkrua, Yoshimitsu Akiyama, Yutaka Hashimoto, Takeshi Otsubo, and Yasuhito Yuasa1. MiR-9 down-regulates CDX2 expression in gastric cancer cells. *Int J Cancer*, Jan 2011. doi: 10.1002/ijc.25923.
29. Daniel Schraivogel, Lasse Weinmann, Dagmar Beier, Ghazaleh Tabatabai, Alexander Eichner, Jia Yun Zhu, Martina Anton, Michael Sixt, Michael Weller, Christoph P Beier, and Gunter Meister. CAMTA1 is a novel tumour suppressor regulated by miR-9/9(*) in glioblastoma stem cells. *EMBO J*, Aug 2011. doi: 10.1038/emboj.2011.301.
30. S Duygu Selcuklu, Mark T A Donoghue, Kristina Rehmet, Matheus de Souza Gomes, Antoine Fort, Prasad Kovvuru, Mohan K Muniyappa, Michael J Kerin, Anton J Enright, and Charles Spillane. Microrna-9 inhibition of cell proliferation and identification of novel mir-9 targets by transcriptome profiling in breast cancer cells. *J Biol Chem*, 287(35):29516–28, Aug 2012. doi: 10.1074/jbc.M111.335943.
31. Reut Shalgi, Daniel Lieber, Moshe Oren, and Yitzhak Pilpel. Global and local architecture of the mammalian microRNA-transcription factor regulatory network. *PLoS Comput Biol*, 3(7):e131, Jul 2007. doi: 10.1371/journal.pcbi.0030131.
32. Jiayi Wang, Zhidong Gu, Peihua Ni, Yongxia Qiao, Changqiang Chen, Xiangfan Liu, Jiafei Lin, Ning Chen, and Qishi Fan. NF-kappaB P50/P65 hetero-dimer mediates differential regulation of CD166/ALCAM expression via interaction with microRNA-9 after serum deprivation, providing evidence for a novel negative auto-regulatory loop. *Nucleic Acids Res*, 39(15):6440–55, Aug 2011. doi: 10.1093/nar/gkr302.
33. Jin Wang, Martin Haubrock, Kun-Ming Cao, Xu Hua, Chen-Yu Zhang, Edgar Wingender, and Jie Li. Regulatory coordination of clustered microRNAs based on microRNA-transcription factor regulatory network. *BMC Syst Biol*, 5(1):199, Dec 2011. doi: 10.1186/1752-0509-5-199.
34. Kun Wang, Bo Long, Jing Zhou, and Pei-Feng Li. miR-9 and NFATc3 regulate myocardin in cardiac hypertrophy. *J Biol Chem*, 285(16):11903–12, Apr 2010. doi: 10.1074/jbc.M109.098004.
35. Xiaowei Wang and Xiaohui Wang. Systematic identification of microRNA functions by combining target prediction and expression profiling. *Nucleic Acids Res*, 34(5):1646–52, 2006. doi: 10.1093/nar/gkl068.
36. Ying Wang, Xiaoman Li, and Haiyan Hu. Transcriptional regulation of co-expressed microRNA target genes. *Genomics*, 98(6):445–52, Dec 2011. doi: 10.1016/j.ygeno.2011.09.004.
37. Zhen Wang, Melis Kayikci, Michael Briese, Kathi Zarnack, Nicholas M Luscombe, Gregor Rot, Blaž Zupan, Tomaž Curk, and Jernej Ule. iCLIP predicts the dual splicing effects of TIA-RNA interactions. *PLoS Biol*, 8(10):e1000530, 2010. doi: 10.1371/journal.pbio.1000530.
38. Xueping Yu, Jimmy Lin, Donald J Zack, Joshua T Mendell, and Jiang Qian. Analysis of regulatory network topology reveals functionally distinct classes of microRNAs. *Nucleic Acids Res*, 36(20):6494–503, Nov 2008. doi: 10.1093/nar/gkn712.
39. Jenny Zhang, Dereje D Jima, Cassandra Jacobs, Randy Fischer, Eva Gottwein, Grace Huang, Patricia L Lugar, Anand S Lagoo, David A Rizzieri, Daphne R Friedman, J Brice Weinberg, Peter E Lipsky, and Sandeep S Dave. Patterns of microRNA expression characterize stages of human B-cell differentiation. *Blood*, 113(19):4586–94, May 2009. doi: 10.1182/blood-2008-09-178186.
40. Jianhu Zhang, Vishnu Chintalgattu, Tiffany Shih, Di Ai, Ying Xia, and Aarif Y Khakoo. MicroRNA-9 is an activation-induced regulator of PDGFR-beta expression in cardiomyocytes. *J Mol Cell Cardiol*, 51(3):337–46, Sep 2011. doi: 10.1016/j.yjmcc.2011.05.019.

SUPPLEMENTARY FIGURES

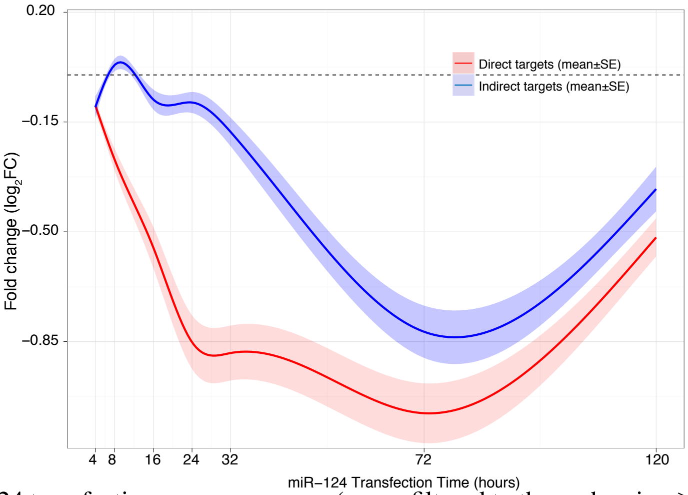

Supplementary Figure 1: miR-124 transfection response curves (genes filtered to those showing ≥ 1.4 FC down–regulation at any time point). (data from [35]; figure based on [25]).

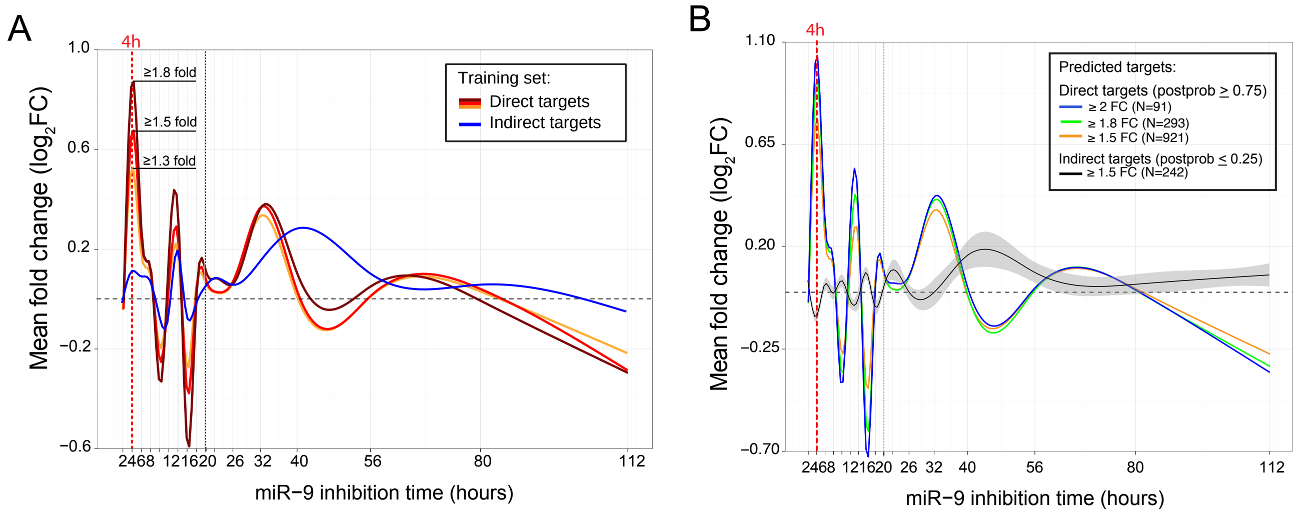

Supplementary Figure 2: Direct and indirect target response curves of gene expression fold change (FC) following miR-9 inhibition.

(A) Red curves show the mean (log2) FC response for a set of genes computationally enriched for direct miRNA target genes (used for the training sets in the predictive models, see Methods). This shows an initial direct inhibition response by 4 hours and downstream responses. For comparison, direct target response is shown for genes with a minimal response of $\geq$ 1.8-fold, 1.5-fold, and 1.3-fold for brown, red and orange respectively. By contrast, the blue curve shows mean FC for a set of of genes enriched for indirect miR-9 target genes and does not show a substantial 4 hour response.

(B) Mean response curves of high confidence predicted direct targets ($\geq$0.75 posterior probability) with mean FC at various fold change thresholds at 4 hours ($\geq$1.5 to $\geq$2-fold) versus predicted indirect targets ($\leq$0.25 posterior probability = black). Predicted direct miR-9 targets show an early 4 hour peak and a 32 hour up–regulatory coherent response, compared with no substantial response for predicted indirect miR-9 targets (black).

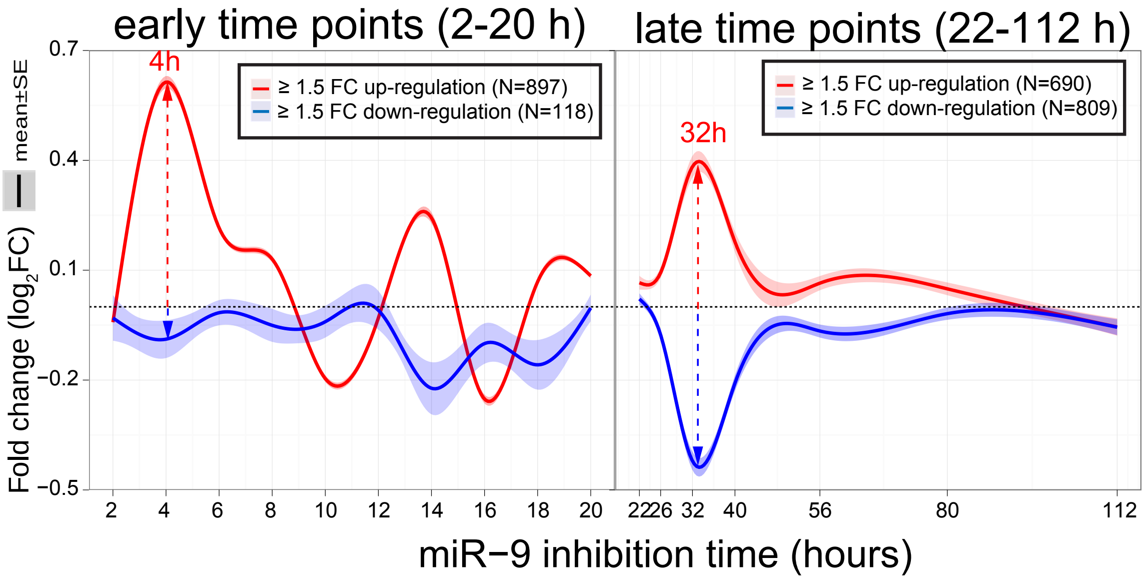

Supplementary Figure 3: Time–series response curves of gene expression fold change (FC) following miR-9 inhibition. Mean FC curves of up-regulated genes (red) and down-regulated genes (blue), absolute FC ≥ 1.5 at any time point, are shown. Early time points (2 to 20 hours: N_{up}=897, N_{down}= 118), and later time points (22 to 112 hours: N_{up}=690, N_{down}= 809) are shown on separate x–axes, with time scale of early time points expanded to show details. The up-regulated gene response curve shows a pronounced asymmetry with an upward peak at 4 hours (log2 FC = 0.62) and 32 hours (log2 FC = 0.49), compared with the down-regulated gene response curve with substantial downward peak at 32 hours only (log2 FC 4 hours = -0.09; log2 FC 32 hours = -0.42). This demonstrates statistically significant asymmetry at 4 hours (p=3E-48; Wilcoxon test) but not at 32 hours (p=0.17; Wilcoxon test). (band shows SEM at each curve position).

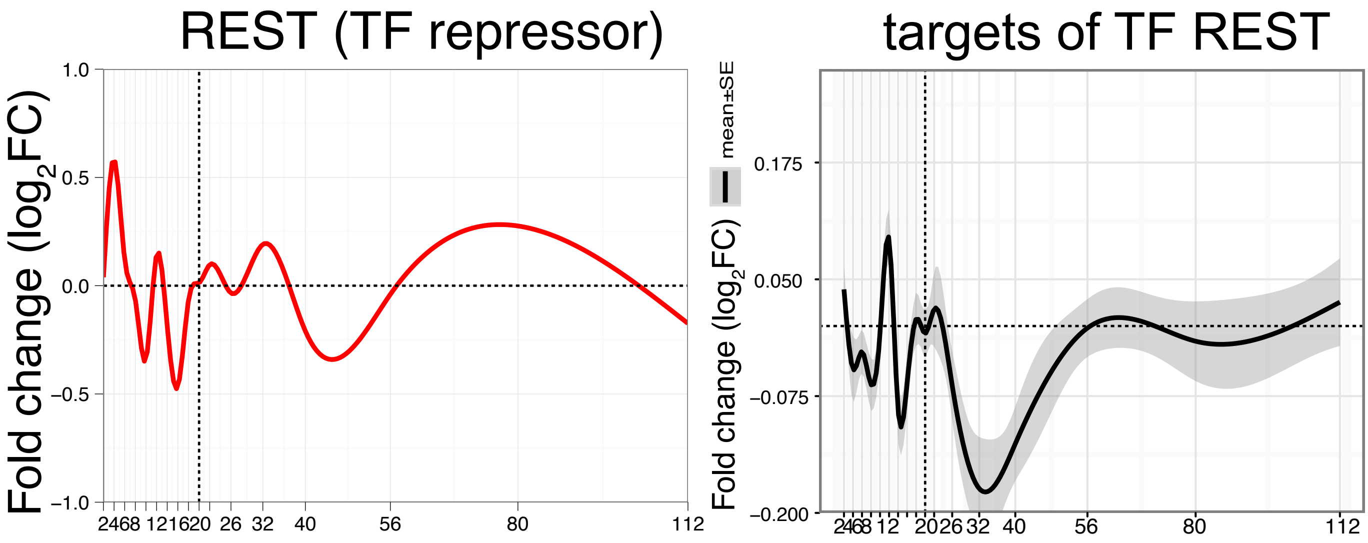

Supplementary Figure 4: Mean FC response curves over transcription factor REST targets. Red: TF gene response; Black: mean TF target genes response. TF gene *REST* is a predicted miR-9 direct target and a known transcriptional repressor. Consistent with being a transcriptional repressor, the mean curve of its target genes shows a negative response at 32 hours. The transcription factor REST (aka NRSF) is a candidate component in a coherent feed–back mechanism involving miR-9. It is involved in validated examples of positive feedback involving miR-9 in other tissues. Notably, it is a predicted miR-9 direct target and is known to regulate miR-9 promoters to cause transcriptional gene silencing by histone acetylation; miR-9-1 and miR-9-2 promoters have been reported as REST targets in human, and all three paralogs in mouse [4, 14, 24]. This feedback cycle could hypothetically lead to a coherent upward response downstream after miR-9 inhibition, with the initial de-repression of miR-9 target *REST* leading to a later, approximately 32 hour, amplified de-repression of miR-9 targets due to transcriptional REST repression of miR-9 genes. Grey bands are 1 SEM. Analysis restricted to expressed genes showing ≥ 1.5 FC at some time point. TF target genes computationally predicted by promoter motif search [17].

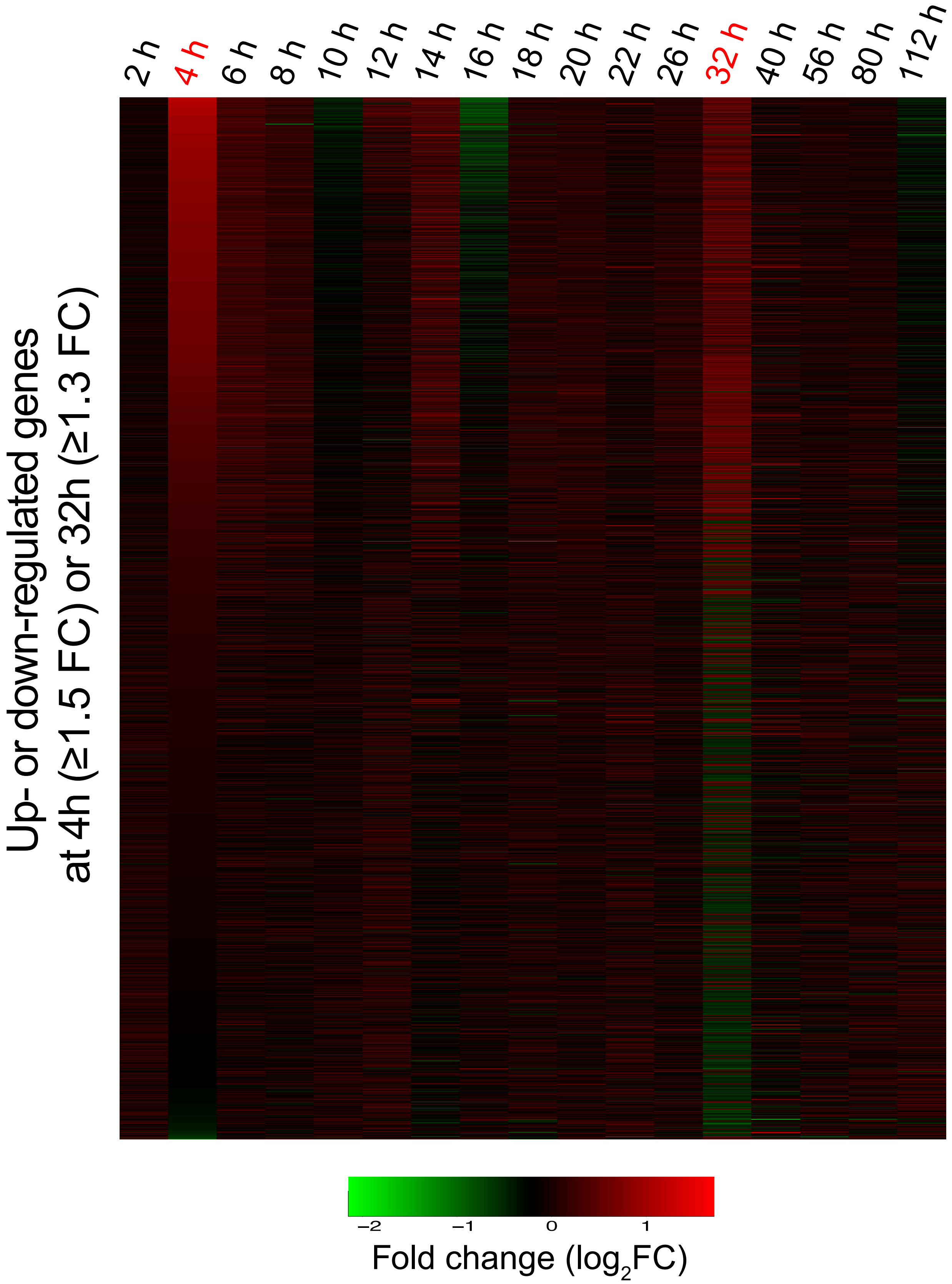

Supplementary Figure 5: Heatmap showing the distribution of genes with substantial FC response at 4 or at 32 hours (absolute FC $\geq$ 1.5 at 4 hr or $\geq$ 1.3 at 32 h), highlighting that substantial 4 hour responses tend to be followed by upward responses at 32 hours. (red = up-response; green = down–response; rows sorted by 4 hour response).

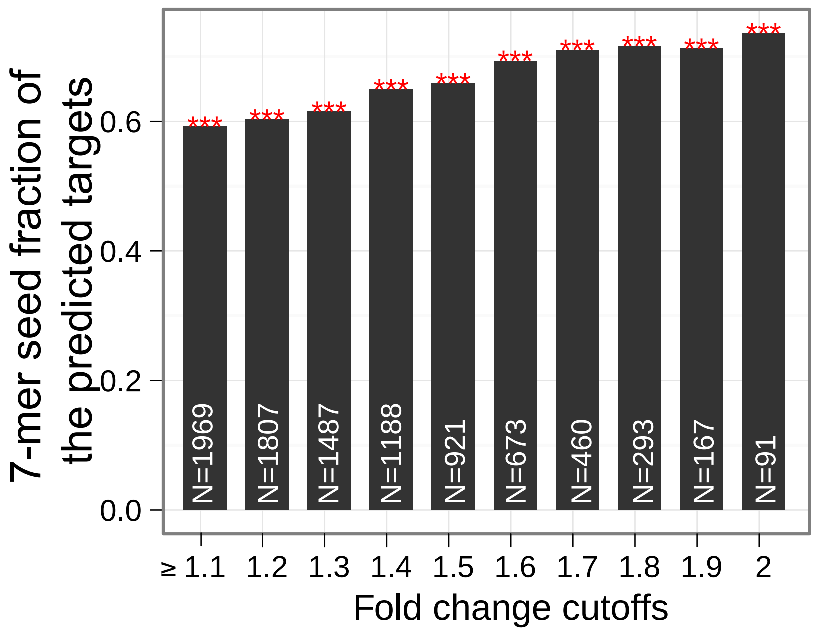

Supplementary Figure 6: 7-mer miR-9 seed enrichment in UTR/CDS of predicted direct targets at 4 hours for varying FC threshold. y-axis shows 7-mer seed fraction of the predicted targets, statistical significance marked as red stars (P <1E-3).

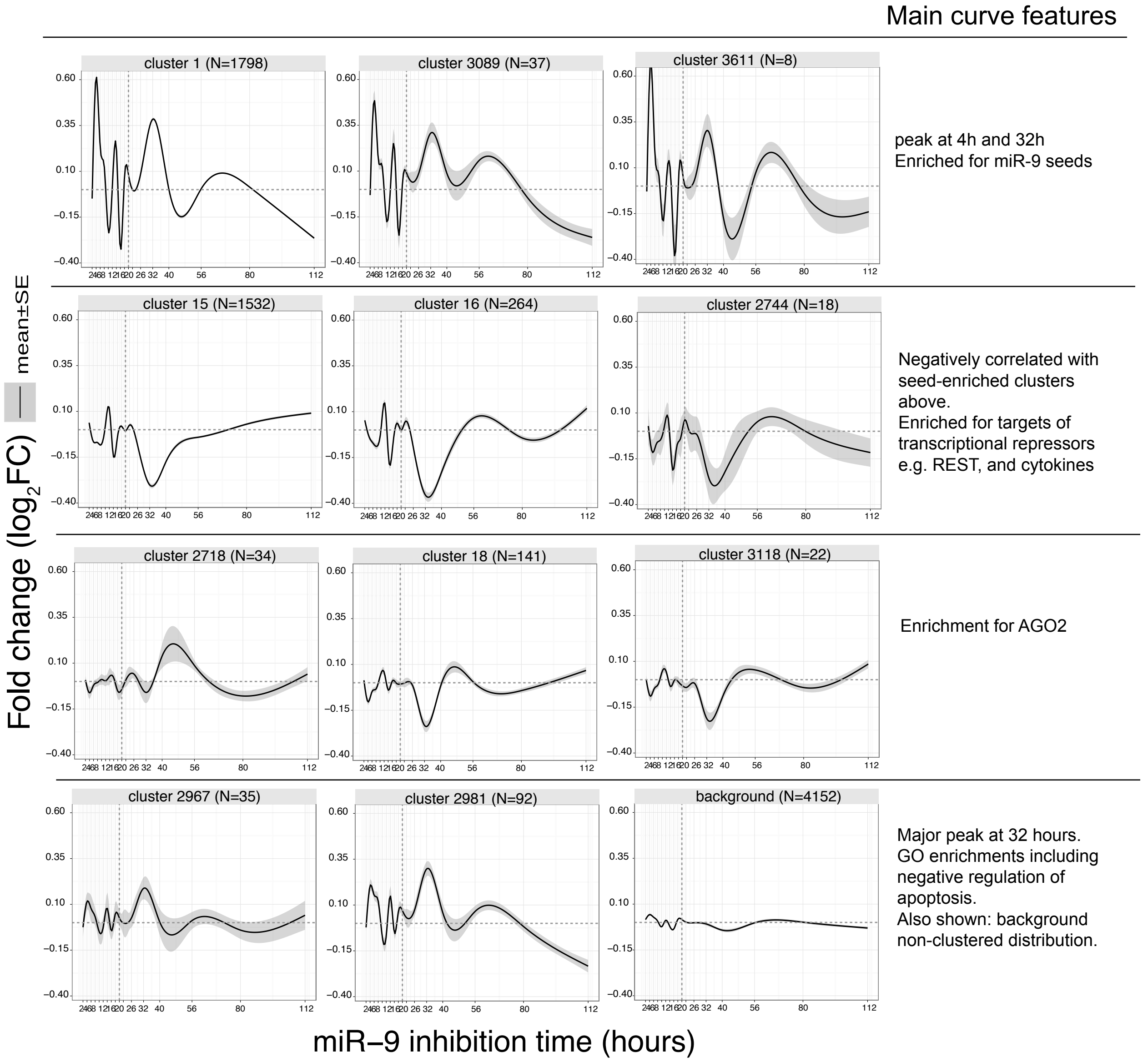

Supplementary Figure 7: Functional clustering results for miR-9 LNA inhibition data. (A) Mean FC curves for functional clusters, broadly classified by major curve features. Cluster 1 is highly enriched for direct miR-9 targets. Bands show SEM at each curve position. The major curve features are described at right.

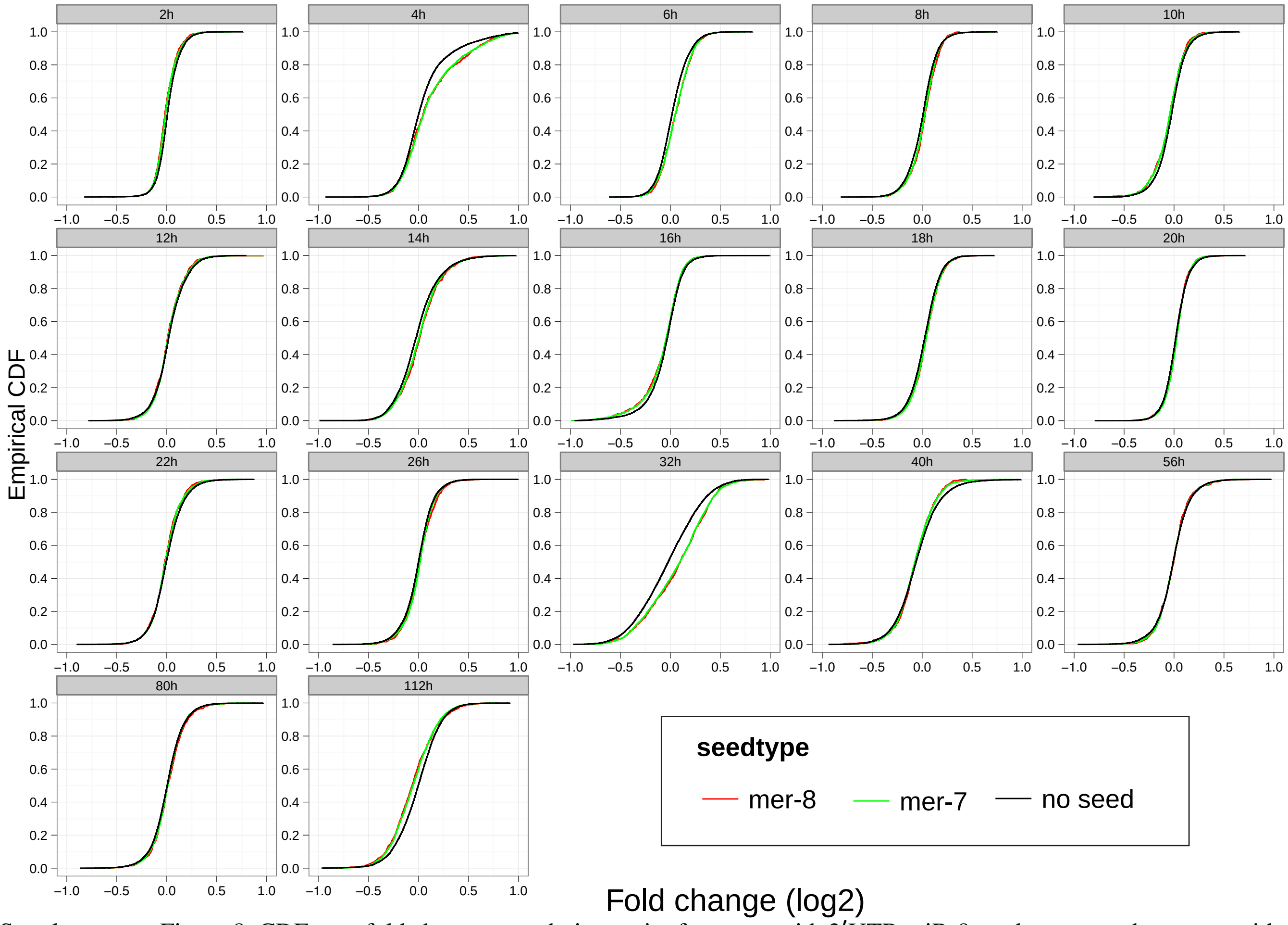

Supplementary Figure 8: CDF over fold change at each time point for genes with 3′UTR miR-9 seeds compared to genes without 3′UTR miR-9 seeds. At 4 hours, genes with miR-9 seeds show substantial up-regulation relative to control genes.

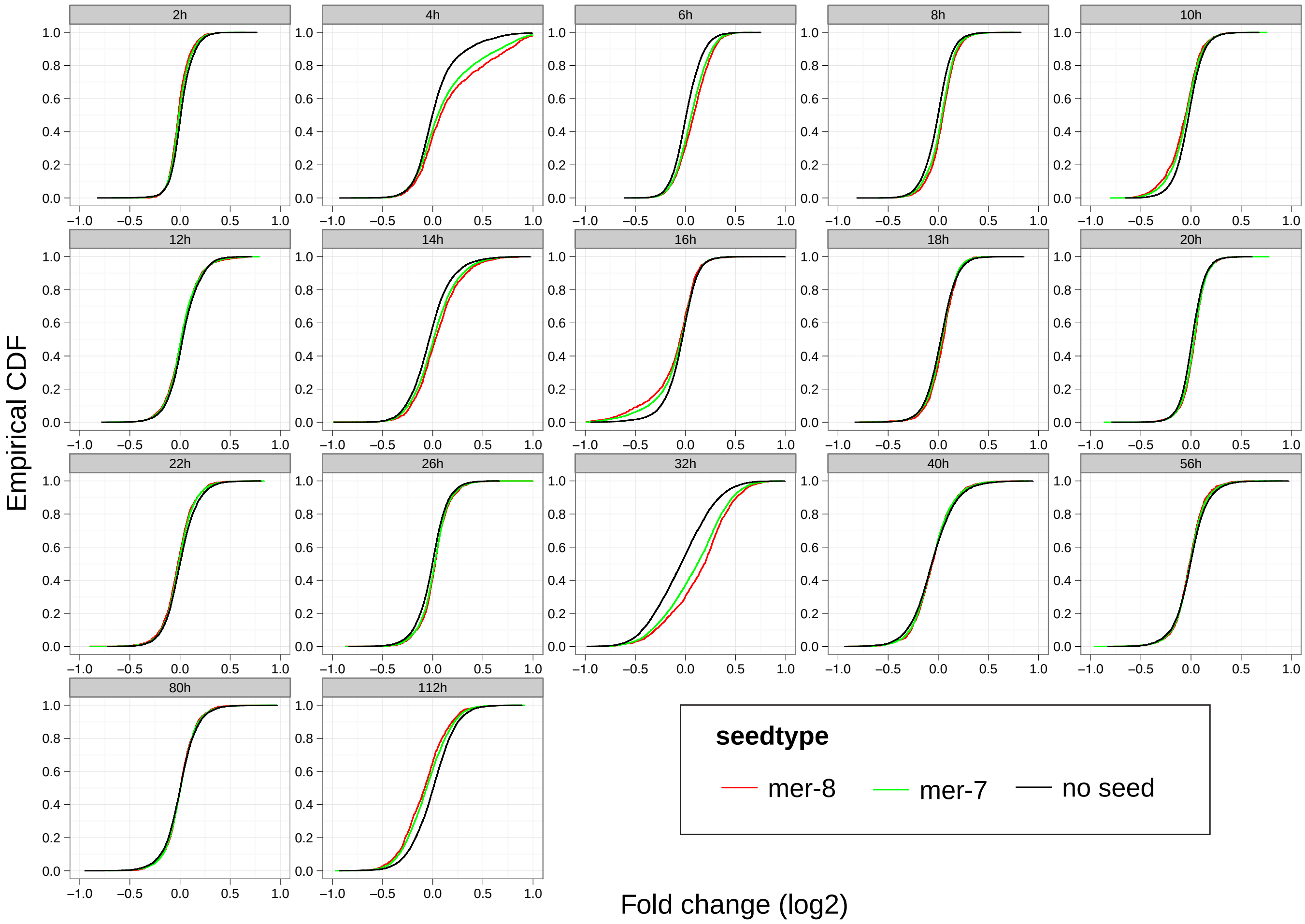

Supplementary Figure 9: CDF over fold change at each time point for genes with CDS miR-9 seeds compared to genes without CDS miR-9 seeds. At 4 hours, genes with miR-9 seeds show substantial up-regulation relative to control genes.

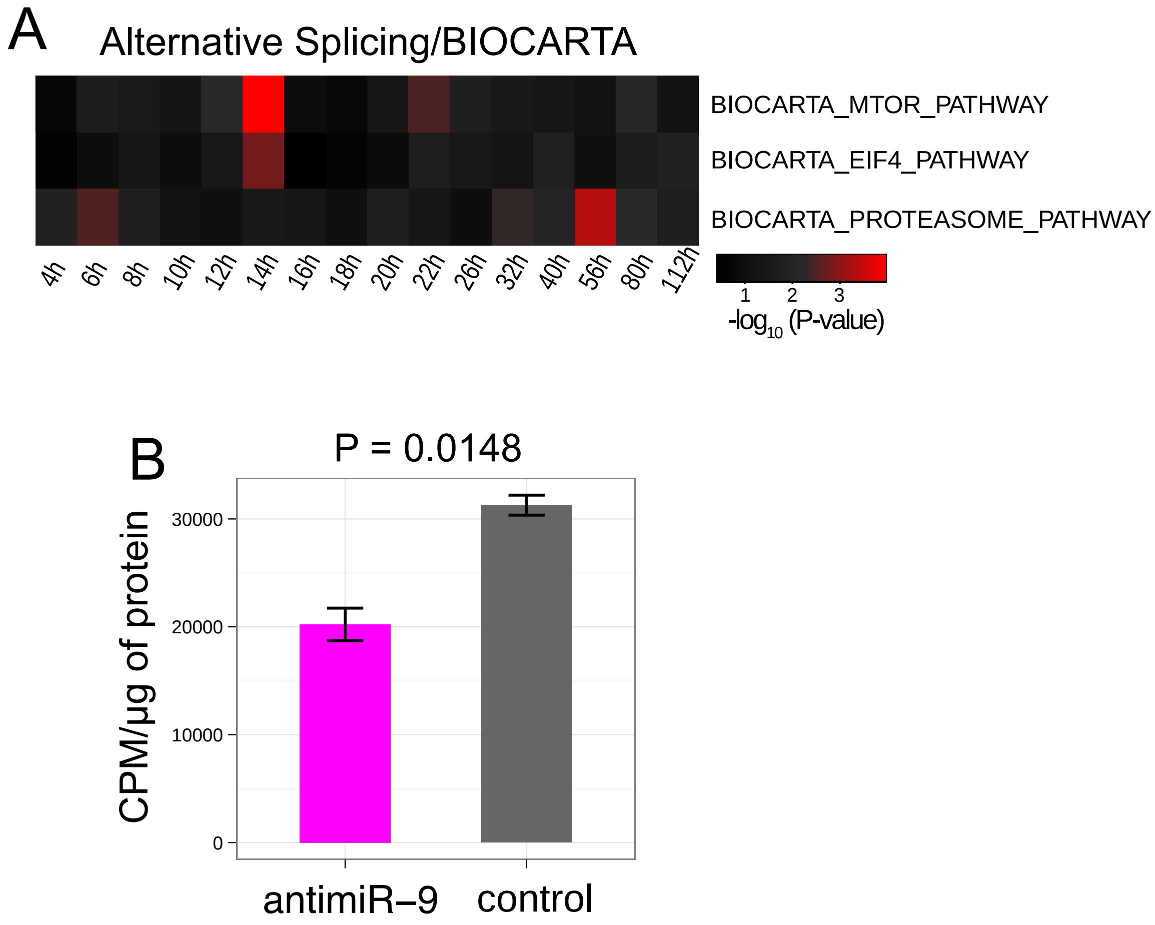

Supplementary Figure 10: (A) GSEA enrichment based on alternative splicing (Biocarta pathways; FDR < 0.05). The mTOR translational control pathway particularly shows high enrichment downstream at 14 hours. Dynamic changes in alternative splicing patterns were analysed by applying the FIRMAGene alternative–splicing R package [27] to LNA versus control microarray files, separately at each time point. Geneset enrichment analysis was performed based on the resulting gene alternative splicing statistic for the genesets of the Biocarta pathways database. (B) Metabolic labelling assay to measure protein production following the miR-9 inhibition. Y-axis is counts per minutes (CPM) normalised to total protein load and error bars show standard error of 3 biological replicates (p=0.0148; t-test). Decreased protein metabolism following miR-9 inhibition relative to controls is consistent with mTOR pathway perturbation. Changes in global translational rates were measured by monitoring the incorporation of 35S-labeled methionine (PerkinElmer), as previously described [3]. Metabolic labelling of L428 cells used 35S methionine. L428 cells were treated with a miR-9 inhibitor or control (3 biological replicates). Cells were assayed 24 hours after transfection. Counts per minute (CPM) were normalised to total protein load.

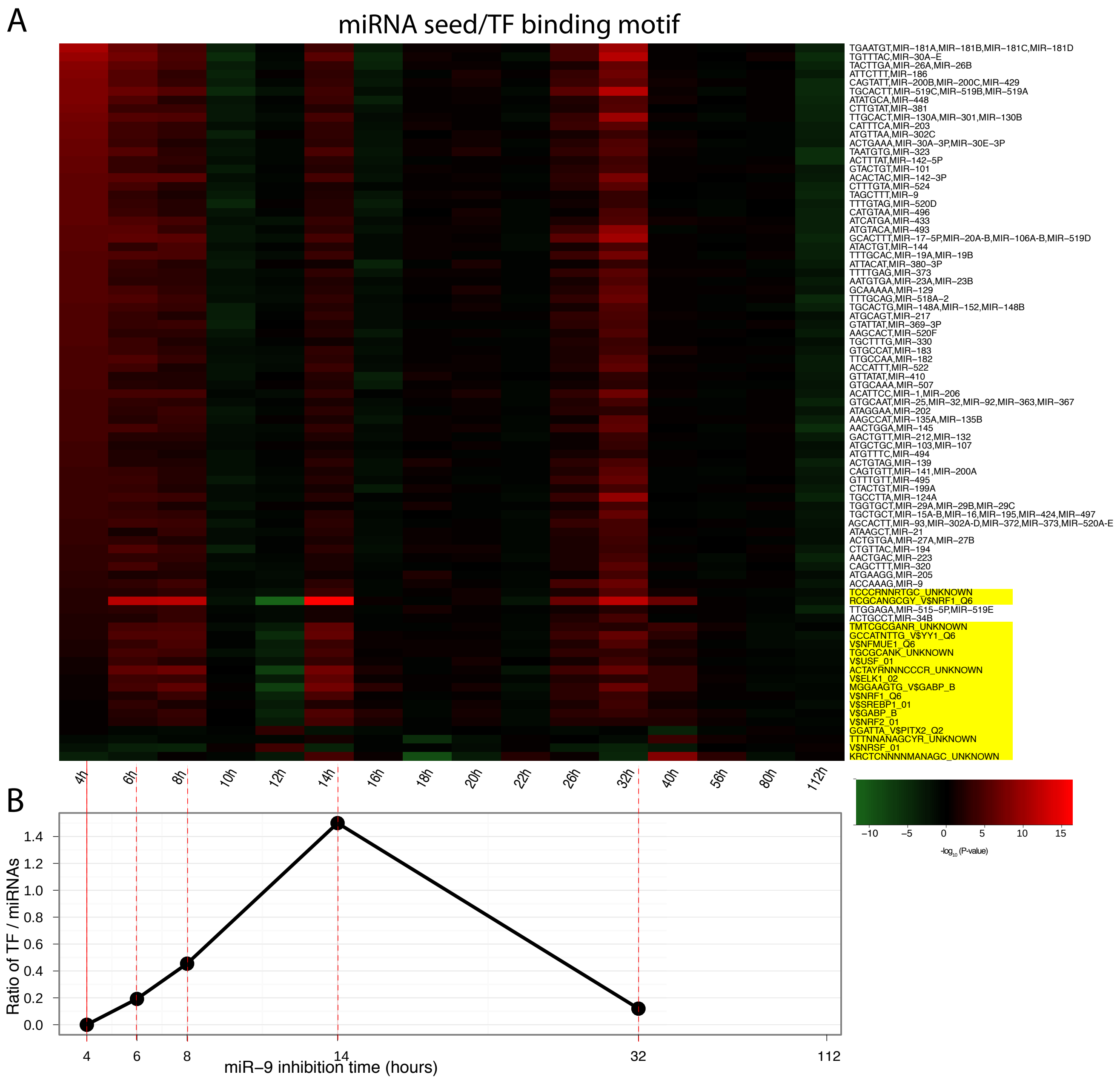

Supplementary Figure 11: Heatmap showing interaction of miR-9 seeds and TF motifs. Red shows statistical enrichment of TF motifs and miRNA seeds in up–regulated genes, green shows enrichment in down—regulated genes. Rows ordered by increasing 4 hour P-value. (A) TF and miRNA seed motif enrichment. Terms listed show significant P-value (< 1E-4) at some time point. TF motifs highlighted in yellow. Several miRNA families as well as miR-9 showed statistically significant enrichment: we hypothesise that these represent co–targetting of miR-9 target genes by these other miRNAs in HL, as well as potential confounding by an increased overall 3′UTR length of miR-9 targets. It has been noted that genes that are regulated by one family of miRNA are often combinatorially regulated by other families of miRNAs (target "hub" genes) [2, 7, 31, 33, 36, 38]. Indeed, we found that those genes with seeds matching these statistically enriched miRNA families also showed a significantly higher overlap with miR-9 target genes compared with other miRNAs (p=1E-5).

(B) Ratio of number of significant TF to miRNA motifs across time. At 4 hours are significantly enriched miRNA seed motifs but no significant TF motifs; 14 hours shows substantial enrichment of TF motifs relative to miRNA seeds.

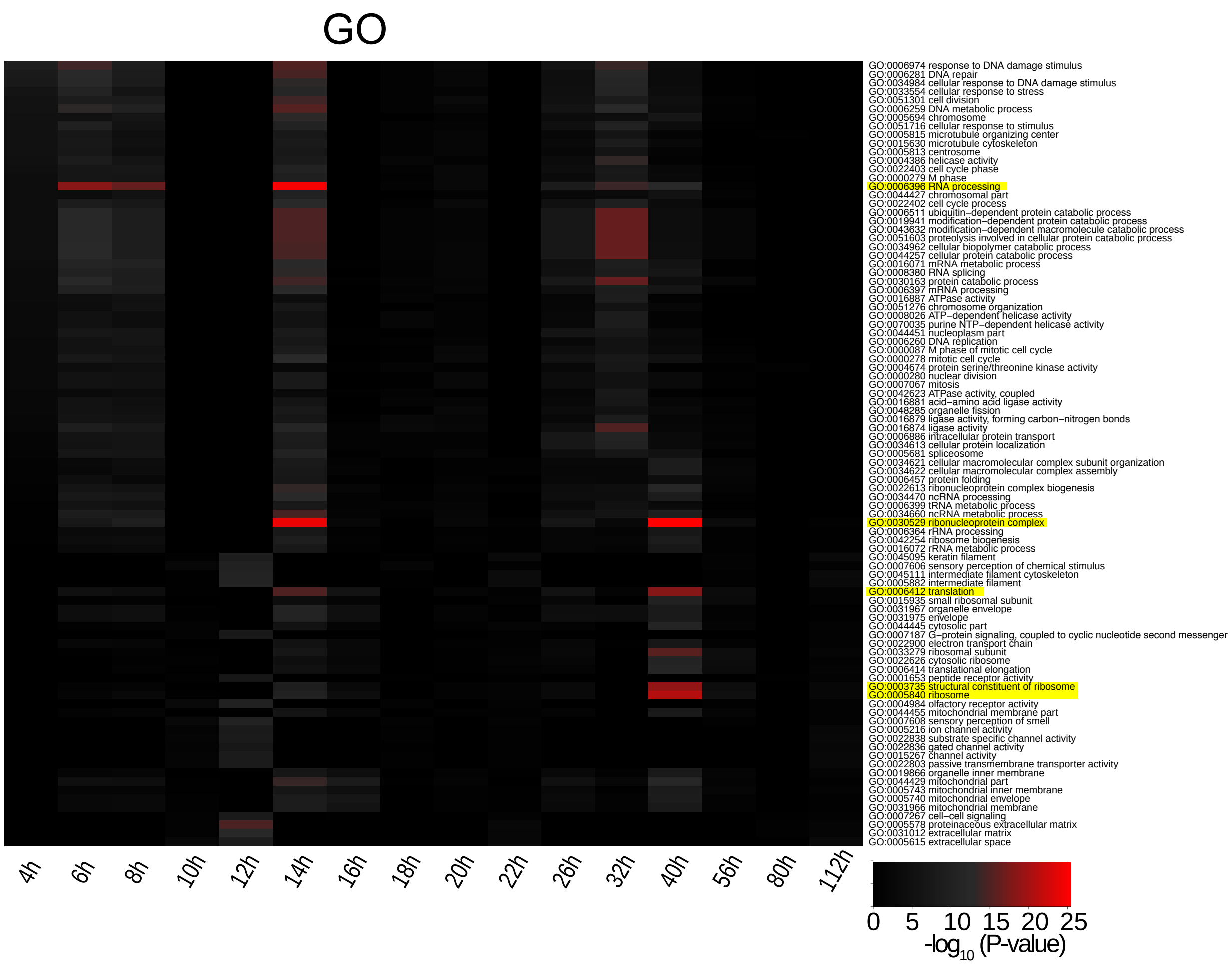

Supplementary Figure 12: Heatmap showing GO term enrichment. Terms listed show significant up–regulation (P-value < 1E-4) at some time point. Terms discussed in the main text (with P < 1E-7) are highlighted in yellow. The most highly significant terms show RNA processing at early time points (6–8 hours) and translation and ribosomal–related terms at later time points (approx. 40 hours), and protein catabolic terms at 32 hours. 14 hours shows enrichment of both RNA processing and ribonucleoprotein GO terms. 12 hours shows significant enrichment for extracellular matrix terms.

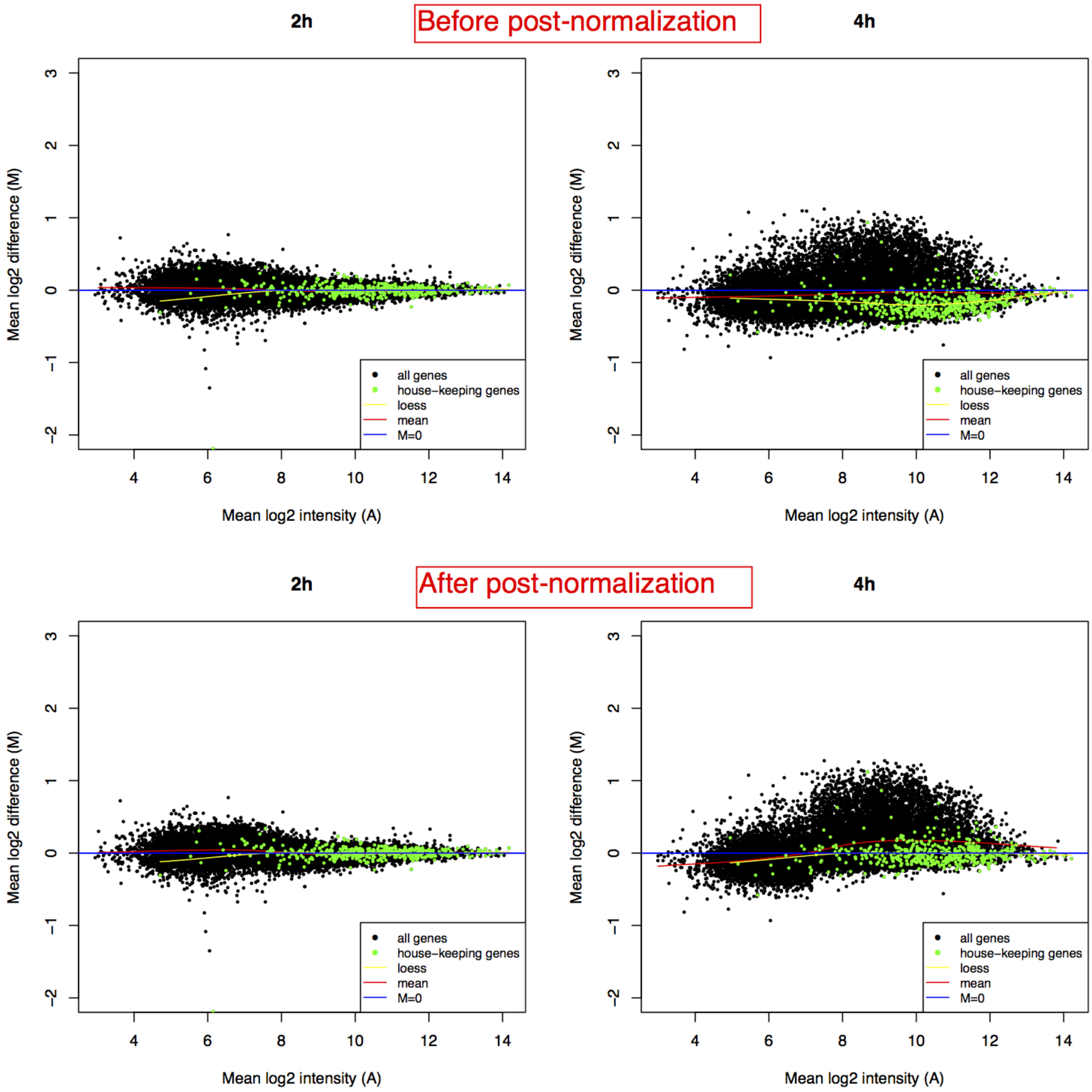

Supplementary Figure 13: MA plots before and after additional post–normalisation step based on Loess correction to housekeeping gene controls. Upper plots show before post–normalisation; lower plots show after post–normalisation. At 4 hours, the increase in positive fold change of many direct target genes leads to housekeeping genes (green) showing a systematic shift downwards (yellow line, upper plot) before correction, due to suboptimal performance of RMA normalisation under these conditions. The lower plot at 4 hours shows the house–keeping gene controls are correctly centred at 0 after adjustment.

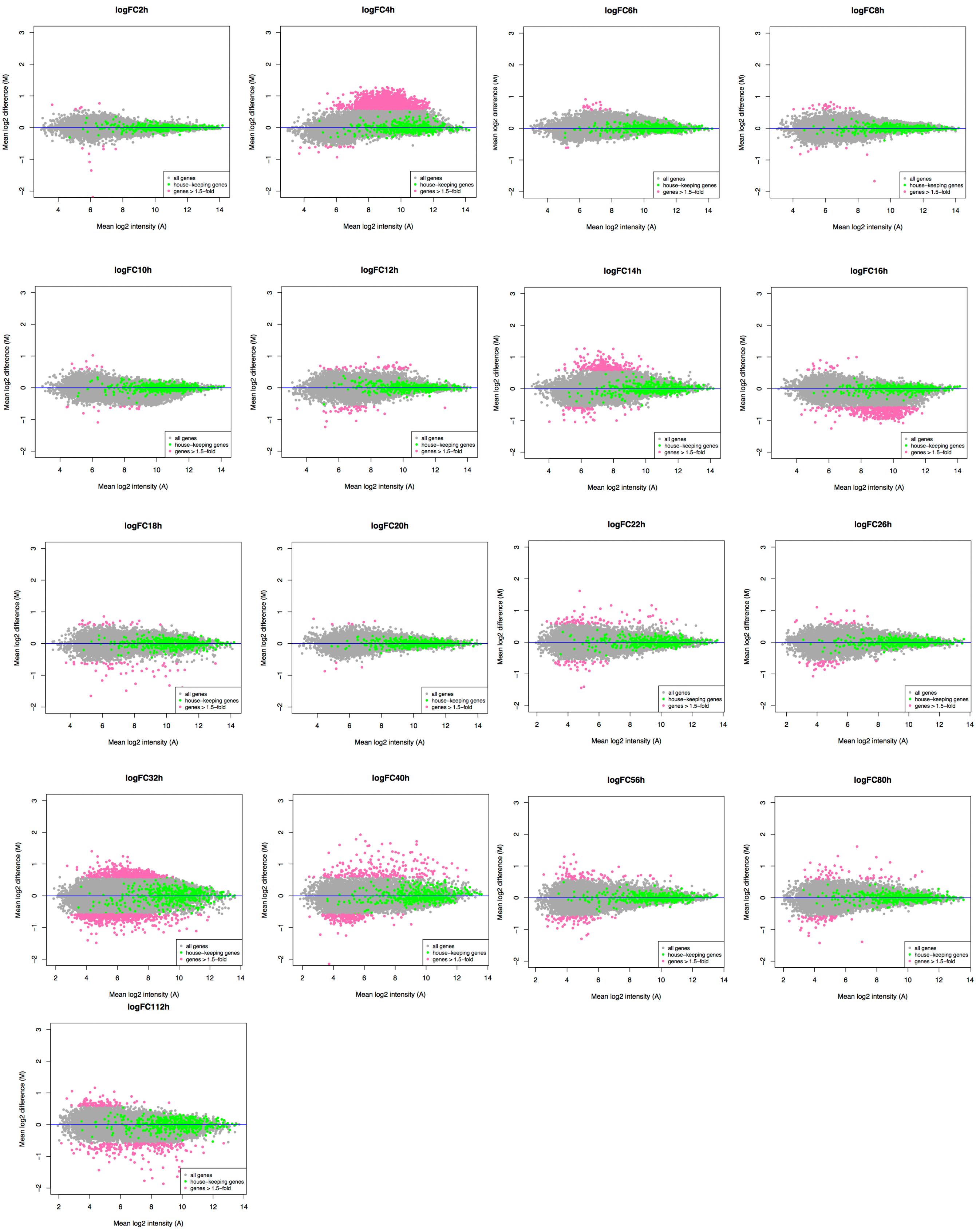

Supplementary Figure 14: MA plots (2h–112h)
